# ADSC secretome constrains NK cell activity by attenuating IL-2-mediated JAK-STAT and AKT signaling pathway via upregulation of CIS and DUSP4

**DOI:** 10.1186/s13287-023-03516-z

**Published:** 2023-11-14

**Authors:** Eunhee Ko, Taejun Yoon, Yoojin Lee, Jongsun Kim, Yong-Beom Park

**Affiliations:** 1 Division of Rheumatology, Department of Internal Medicine, Yonsei University College of Medicine, Seoul, 03722 Republic of Korea; 2 Brain Korea 21 PLUS Project for Medical Science, Yonsei University College of Medicine, Seoul, 03722 Republic of Korea; 3 Institute for Immunology and Immunological Diseases, Yonsei University College of Medicine, Seoul, 03722 Republic of Korea; 4 Department of Microbiology, Yonsei University College of Medicine, Seoul, 03722 Republic of Korea

**Keywords:** Mesenchymal stem cells, Secretome, Immunomodulation, Natural killer cells, CD96, Cytokine-inducible SH2-containing protein, Dual specificity phosphatase 4, Proteomics, Transcriptomics

## Abstract

**Background:**

Mesenchymal stem cells (MSCs) have immunomodulatory properties and therapeutic effects on autoimmune diseases through their secreted factors, referred to as the secretome. However, the specific key factors of the MSC secretome and their mechanisms of action in immune cells have not been fully determined. Most in vitro experiments are being performed using immune cells, but experiments using natural killer (NK) cells have been neglected, and a few studies using NK cells have shown discrepancies in results. NK cells are crucial elements of the immune system, and adjustment of their activity is essential for controlling various pathological conditions. The aim of this study was to elucidate the role of the adipose tissue-derived stem cell (ADSC) secretome on NK cell activity.

**Methods:**

To obtain the ADSC secretome, we cultured ADSCs in medium and concentrated the culture medium using tangential flow filtration (TFF) capsules. We assessed NK cell viability and proliferation using CCK-8 and CFSE assays, respectively. We analyzed the effects of the ADSC secretome on NK cell activity and pathway-related proteins using a combination of flow cytometry, ELISA, cytotoxicity assay, CD107a assay, western blotting, and quantitative real-time PCR. To identify the composition of the ADSC secretome, we performed LC–MS/MS profiling and bioinformatics analysis. To elucidate the molecular mechanisms involved, we used mRNA sequencing to profile the transcriptional expression of human blood NK cells.

**Results:**

The ADSC secretome was found to restrict IL-2-mediated effector function of NK cells while maintaining proliferative potency. This effect was achieved through the upregulation of the inhibitory receptor CD96, as well as downregulation of activating receptors and IL-2 receptor subunits IL-2Rα and IL-2Rγ. These changes were associated with attenuated JAK-STAT and AKT pathways in NK cells, which were achieved through the upregulation of cytokine-inducible SH2-containing protein (CIS, encoded by *Cish*) and dual specificity protein phosphatase 4 (DUSP4). Furthermore, proteomic analysis revealed twelve novel candidates associated with the immunomodulatory effects of MSCs.

**Conclusions:**

Our findings reveal a detailed cellular outcome and regulatory mechanism of NK cell activity by the ADSC secretome and suggest a therapeutic tool for treating NK-mediated inflammatory and autoimmune diseases using the MSC secretome.

**Supplementary Information:**

The online version contains supplementary material available at 10.1186/s13287-023-03516-z.

## Background

Natural killer (NK) cells are large granular lymphocytes that induce apoptosis in target cells by secreting cytotoxic granules, such as perforin and granzyme B, through exocytosis without prior sensitization [1]. In addition, they rapidly secrete several cytokines and chemokines, such as IFN-γ, GM-CSF, IL-10, TNF-α, and CCL3-5, to induce or amplify the inflammatory response through various mechanisms [2, 3]. NK cell activation is primarily controlled by strict engagement and balance between activating and inhibitory surface receptors, which transduce each signal through their immunoreceptor tyrosine-based activation motifs (ITAMs) or inhibition motifs (ITIMs) in their cytoplasmic tail [4, 5], and by the integration of signals from cytokines, such as IL-2, IL-12, IL-15, and IL-18 [6, 7]. Activating receptors recognize pathogen-derived or stress-induced ligands and induce cytokine secretion and cytotoxicity to kill the target cells. Conversely, inhibitory receptors recognize the major histocompatibility complex (MHC) class I present on healthy host cells, thereby protecting healthy cells from NK cell-mediated killing [8, 9].

When NK inhibitory receptors bearing ITIMs engage their ligands, there is a recruitment of protein tyrosine phosphatases (PTPs) such as Src homology 2 (SH2) domain-containing protein tyrosine phosphatases (SHP-1 and SHP-2) and SH2 domain-containing inositol phosphatases (SHIP-1 and SHIP-2), which dephosphorylate signaling proteins linked to activation signals, resulting in the suppression of NK cell responses [5, 10]. Moreover, these PTPs also modulate the cytokine-mediated Janus kinase (JAK)-signal transducer and activator of transcription (STAT) pathway together with suppressor of cytokine signaling (SOCS) proteins, which act as a negative-feedback loop and are induced by cytokines to block further signal transduction [11, 12]. They cause target proteins (tyrosine-phosphorylated JAKs or STATs) to undergo proteasomal degradation via ubiquitination [13]. Compromising NK cell activity may cause increased susceptibility to infection and cancer, whereas inordinate NK cell activity may be an occasion of severe tissue damage [14, 15]. Although adjusting NK cell activity is essential for controlling various pathological conditions, the fundamental endogenous mechanisms for modulating the functional outcome of NK cell activity remain to be investigated.

Mesenchymal stem cells (MSCs) have versatile biological properties depending on their cellular microenvironment, where they promote tissue regeneration and immune regulation [16]. Many researchers have focused on harnessing their therapeutic capabilities to treat degenerative and inflammatory disorders. It is currently accepted that the immunomodulatory property of MSCs is due to their secreted factors, collectively referred to as the secretome, acting in a paracrine manner rather than the cells themselves [17–19]. Current attention has shifted to secretome-based therapy for clinical applications because it could be considered as an alternative to cell-based therapy [20–22].

The MSC secretome consists of various molecules, such as cytokines, chemokines, nucleic acids, growth factors, proteases, microvesicles, exosomes, and immune modulatory factors. This composition of the MSC secretome is manipulated to improve therapeutic effects by some strategies, such as priming using biological stimuli and genetic modification via transfection or transduction [19, 21]. The MSC secretome is known to possess therapeutic benefits through its immunomodulatory, anti-inflammatory, anti-apoptotic, and proangiogenic properties [23, 24]. Previous studies have reported that the MSC secretome contains a variety of bioactive factors capable of regulating proliferation, differentiation, and effector function of immune cells, including NK cells, thereby exerting therapeutic effects in inflammatory and autoimmune diseases [25, 26].

For this reason, there is accumulating evidence revealing the precise composition with therapeutic potential in the MSC secretome through common techniques including proteomic analysis for clinical applicability [27–36]. However, numerous studies have not fully elucidated the specific key factors of the MSC secretome that mediate the outcome in immune cells or tissues, demonstrating the difficulty of accurately determining which factors within the MSC secretome are responsible for immunoregulation [19]. Therefore, studies characterizing MSC-secreted factors precisely and identifying the major factors responsible for immunomodulation in the secretome and their mechanisms of action are essential for the development of advanced therapies using stem cells [37]. In order to reach that point, most in vitro experiments use T cells, B cells, and macrophages, but only a few use natural killer (NK) cells [38]. To date, even a few studies on the effect of MSC-derived soluble factors on NK cells [39–43] have shown discrepancies. Therefore, it is necessary to elucidate how the MSC secretome affects NK cells, and the precise molecular mechanisms underlying these effects.

To address these questions, we accounted for a few aspects of secretome effects in the context of the overall cellular phenomena and the expression changes of intracellular signaling molecules using a human NK cell line and blood-derived NK cells. In this study, we demonstrated that the secretome from human adipose tissue-derived stem cells (ADSCs) constrained IL-2-mediated effector function of NK cells, including cytokine production and target cell killing through the upregulation of inhibitory receptor CD96 along with downregulation of activating receptors and some of the IL-2 receptor subunits, but did not affect cell proliferation. We also found that a negative regulator, CIS, and a phosphatase, DUSP4, related to the IL-2-mediated signaling pathway, were upregulated by the ADSC secretome and identified novel additional candidates associated with immunomodulation of MSCs. Taken together, our findings propose a detailed functional and mechanism-based viewpoint for regulating NK cell activity by the ADSC secretome, supporting a therapeutic tool for treating NK-mediated inflammatory and autoimmune diseases using the MSC secretome.

## Methods

### Cell lines and culture conditions

All cell lines were kindly provided by Prof. Jongsun Kim at Yonsei University College of Medicine. NK-92 cells, a human NK cell line, were cultured in complete growth medium, as indicated in the ATCC’s product sheet (CRL-2407; ATCC, Manassas, VA, USA). Complete growth medium was made with α minimum essential medium (α-MEM; Gibco, Grand Island, NY, USA) supplemented with 0.2 mM inositol (Sigma-Aldrich, St. Louis, MO, USA), 0.1 mM 2-mercaptoethanol (Gibco), 0.02 mM folic acid (Sigma-Aldrich), 1% penicillin/streptomycin (Gibco), 12.5% heat-inactivated fetal bovine serum (FBS; Corning Inc., Corning, NY, USA), 12.5% heat-inactivated horse serum (Gibco), and recombinant human (rh) IL-2 (5 ng/ml; NKMAX Co Ltd., Seongnam, Republic of Korea), essential for cell survival and activation. K562, a human myelogenous leukemia cell line, was cultured in RPMI-1640 medium (Corning Inc.) supplemented with 10% FBS (Corning Inc.), 1% nonessential amino acids (Gibco), 2% HEPES (Corning Inc.), 1% sodium pyruvate (Corning Inc.), 55 μM 2-mercaptoethanol (Gibco), 0.2% gentamycin (Gibco), and 1% penicillin/streptomycin (Gibco). The cells were maintained in a cell incubator at 37 °C and 5% CO_2_.

### Human primary cells and culture conditions

MSCs derived from adipose tissue were used because they are relatively easy to access compared with other tissues such as bone marrow, umbilical cord, and placenta, and have the advantage of being applicable for autologous transplantation. Human ADSCs (PromoCell, Heidelberg, Germany) were expanded in low-glucose Dulbecco’s modified Eagle’s medium (DMEM; Corning Inc.) supplemented with 10% FBS (Corning Inc.), 1% nonessential amino acids (Gibco), and 1% penicillin/streptomycin (Gibco) for five to six passages. To obtain the ADSC secretome, the culture medium was replaced with low-glucose DMEM without phenol red (Gibco), supplemented with 2 mM L-glutamine (Sigma-Aldrich) and 1% penicillin/streptomycin (Gibco) under serum-free conditions. Human primary NK cells were isolated from the human peripheral blood mononuclear cells (PBMCs) of a healthy donor with negative selection using a human NK Cell Isolation Kit (Miltenyi Biotec, Westphalia, Germany) according to the manufacturer’s instructions. All protocols were approved by the Institutional Review Board of Severance Hospital at Yonsei University (IRB no. 4-2020-1062; Date of approval: Nov 9, 2020), and informed consent was obtained after sufficiently explaining the nature and possible consequences of the study to healthy donors. Following isolation, purified NK cells (purity > 90%) were immediately cultured in α-MEM-based complete growth medium with 10 ng/mL rhIL-2 for their survival and activation. The cells were maintained in a cell incubator at 37 °C and 5% CO_2_.

### Generation of human ADSC secretome

ADSCs at passage 5 or 6 (P5 or P6) were dispensed into a total of 100 10-cm culture dishes (Corning Inc.) at a density of 2 × 10^5^ cells/plate and incubated with growth medium in a cell incubator at 37 °C and 5% CO_2_ for expansion to over 90% confluence. After incubation, ADSCs were washed twice with Dulbecco’s phosphate-buffered saline (DPBS) to remove serum and phenol red, replaced with FBS-free medium to generate the secretome, and incubated for 48 h. Thereafter, 1 L of conditioned medium lacking serum and phenol red derived from ADSCs was collected and centrifuged at 1,800‒2,000 rpm for 5 min to remove cell debris or dead cells. This medium was concentrated by ultrafiltration, using a tangential flow filtration (TFF) capsule (Pall Corporation, New York, NY, USA) containing a 3-kDa molecular weight cut-off (MWCO) membrane, according to the manufacturer’s instructions. Subsequently, the ADSC secretome was analyzed using a BCA protein assay (Thermo Fisher Scientific, Waltham, MA, USA) to estimate protein concentration and immediately stored at − 80 °C. All secretomes used in this study had their efficacy verified by screening to determine the secretion level of IFN-γ by NK-92 cells.

### Cell cultures

For cell maintenance, NK-92 cells were dispensed in complete medium in T-25 culture flasks (Thermo Fisher Scientific) at 7 × 10^4^ cells/mL with 5 or 10 ng/mL of rhIL-2 (NKMAX Co Ltd.) at a final volume of 5‒15 mL, and the culture medium was replaced every 2‒3 days depending on cell density. This subculturing procedure was strictly followed because NK-92 cells are highly sensitive to overgrowth and IL-2 depletion. For ADSC secretome studies, NK-92 cells were seeded into 6-well culture plates (Corning Inc.) at 1 × 10^5^ cells/mL and a final volume of 2 mL and stimulated using rhIL-2 (5 ng/mL; NKMAX Co Ltd.). For human primary cells, upon isolation from the blood of a healthy donor, NK cells were plated at a density of 1 × 10^6^ cells/mL in growth medium and dispensed into 24-well culture plates (Corning Inc.) with or without 10 ng/mL of rhIL-2 (NKMAX Co Ltd.).

### Cell counting kit (CCK)-8 assay

Cell viability was measured using a CCK-8 assay kit (Sigma-Aldrich), according to the manufacturer’s instructions. Briefly, 1 × 10^4^ NK-92 cells were seeded in a 96-well flat bottom plate (SPL Life Sciences, Pocheon, Republic of Korea) containing 100 μL medium in triplicate for the accuracy of results, after which they were incubated with or without the ADSC secretome for 48 h. Next, 10 μL of CCK-8 reagent was dispensed into each well, and the plate was incubated for 4 h at 37 °C. Finally, the absorbance was measured at 450 nm using a microplate reader (Molecular Devices, San Jose, Ca, USA).

### Cell proliferation assay

Cell proliferation was assessed using carboxyfluorescein diacetate succinimidyl ester (CFSE; eBioscience, San Diego, CA, USA) according to the manufacturer’s protocol. Briefly, NK-92 cells were labeled with 1 μM of CFSE and then cultured at 1 × 10^5^ cells/mL in a 24-well plate (Corning Inc.) for 48 or 96 h with various concentrations of ADSC secretome. Cells were analyzed on a BD FACSVerse instrument (BD Biosciences, Franklin Lakes, NJ, USA), and data were analyzed using FlowJo v10 software (FlowJo LLC, Ashland, OR, USA).

### Enzyme-linked immunosorbent assay (ELISA)

The concentrations of IFN-γ, IL-10, GM-CSF, perforin, and granzyme B in the culture supernatants were estimated using an ELISA kit (BD Biosciences; R&D Systems, Minneapolis, MN, USA; Mabtech AB, Nacka Strand, Sweden) according to the manufacturer’s instructions. Absorbance was measured at 450 and 570 nm using an ELISA microplate reader (Molecular Devices).

### Cytotoxicity assay

The cytolytic capacity of NK cells against target cells was measured using a Calcein-AM Release Assay (Invitrogen, Carlsbad, CA, USA). Briefly, K562 target cells were stained with 1 μM calcein-AM for 10 min at 37 °C, washed twice with PBS, and resuspended in RPMI complete medium. Next, target cells (1 × 10^4^) in 100 μL medium were seeded in quadruplicate into a round-bottom 96-well plate (Thermo Fisher Scientific) and incubated for 3‒4 h with 100 μL of NK cells at appropriate concentrations to obtain effector-to-target (E:T) ratios. The co-culture supernatant containing calcein-AM released from K562 target cells via lysis was measured using a Varioskan Flash fluorometer (ex/em = 494/517 nm; Thermo Fisher Scientific). The percentage of specific lysis caused by NK cells was calculated according to the following formula:

$$\% \;{\text{specific}}\;{\text{lysis}} = \frac{{{\text{experimental}}\;{\text{release}}_{{{\text{Avg}}}} - {\text{spontaneous}}\;{\text{release}}_{{{\text{Avg}}}} }}{{{\text{maximal}}\;{\text{release}}_{{{\text{Avg}}}} - {\text{spontaneous}}\;{\text{release}}_{{{\text{Avg}}}} }} \times 100$$

where maximal release occurred with the addition of Triton X-100 (final concentration of 2%; Sigma-Aldrich).

### CD107a degranulation assay

NK cell degranulation was measured using PE-conjugated CD107a detection. NK cells pre-treated with or without ADSC secretome for 48 h were seeded into a round bottom 96-well plate (Thermo Fisher Scientific) with 2 × 10^4^ K562 target cells at a final volume of 200 μL and incubated with PE-anti-CD107a antibody (BD Biosciences) at a final dilution of 1:20 for 1 h at 37 °C. Thereafter, GolgiPlug (1,000 × dilution; BD Biosciences) containing brefeldin A, which inhibits intracellular protein transport, was added to each well plate and incubated for 2‒3 h. Afterward, the cells were stained with a PE-Cy7-conjugated CD56 monoclonal antibody for 30 min at 4 °C. CD107a expression in NK cells was analyzed using a BD LSRFortessa X-20 instrument (BD Biosciences), and data were analyzed using FlowJo v10 software (FlowJo LLC).

### Flow cytometry

For surface antigens, the cells were stained with the appropriate monoclonal antibodies (mAbs) for 30 min at 4 °C and washed with PBS supplemented with 5% FBS. For intracellular antigens, cell suspensions were preincubated with phorbol 12-myristate 13-acetate (PMA; 50 ng/mL; Sigma-Aldrich), ionomycin (750 ng/mL; Sigma-Aldrich), and GolgiPlug (1 μg/mL; BD Biosciences) for 4 h. Subsequently, the cells were harvested from in vitro cultures and surface-stained for the indicated markers. After cells were fixed with intracellular (IC) fixation buffer (eBioscience) for 20 min at 18 °C to 25 °C and permeabilized with permeabilization buffer (eBioscience), intracellular cytokine staining was performed using the appropriate monoclonal antibodies for 45 min at 18 °C to 25 °C. Data were collected using a BD LSRFortessa X-20 instrument (BD Biosciences) and analyzed using FlowJo v10 software (FlowJo LLC). Fluorophore-conjugated mAbs against humans were used. BV421-CD56 (HCD56), FITC-CD56 (HCD56), FITC-CD3 (HIT3a), PE-CD56 (HCD56), PE-NKp46 (9E2), PE-CD96 (NK92.39), PE-TIGIT (A15153G), PE-CD94 (DX22), PE-Tim-3 (F38-2E2), PE-IL-10 (JES3-19F1), PE-granzyme B (QA16A02), BV711-CD56 (HCD56), BV711-NKp30 (P30-15), BV711-IFN-γ (4S.B3), BV711-perforin (dG9), PE-Cy7-CD16 (3G8), and PE-Cy7-CD25 (BC96), were obtained from BioLegend. PE-CD107a (H4A3), PE-CD122 (Mik-β2), BV711-NKG2D (1D11), BV711-CD132 (AG184), and PE-Cy7-CD56 (B159) were purchased from BD Biosciences. All fluorophore-conjugated mAbs were diluted at 1:100 or 1:200. Neutralizing antibodies, anti-ANXA1, anti-DKK3, anti-LGALS3BP, anti-PROS1, and anti-PEDF were purchased from Abcam (Cambridge, UK).

### Western immunoblotting

The downstream signaling cascade of IL-2 that induces the survival and activation of NK cells was assessed by probing phosphorylated and total proteins. After collecting NK cells, the cells were washed twice with PBS to remove the residual medium, after which they were lysed in RIPA buffer (Biosesang, Seongnam, Republic of Korea) containing a complete mini protease inhibitor cocktail (Roche Diagnostics, Pleasanton, CA, USA) and PhosSTOP phosphatase inhibitor cocktail (Roche Ciagnostics) and homogenized on ice. Thereafter, the cell lysates were centrifuged at 12,000 rpm for 15 min at 4 °C to remove cell debris. Protein concentrations were determined using a bicinchoninic acid (BCA) protein assay (Thermo Fisher Scientific). Next, equivalent amounts of cellular proteins were separated using sodium dodecyl sulfate (SDS)-polyacrylamide gel electrophoresis (PAGE) and then transferred to methanol-activated polyvinylidene difluoride (PVDF) membranes (Merck Millipore, Darmstadt, Germany). After blocking with block solution (TransLab, Elgin, IL, USA), membranes were probed with primary antibodies for 2 h at 18 °C to 25 °C. Afterward, the membranes were washed six times for 30 min in Tris-buffered saline with Tween 20 (TBS-T), followed by the species-appropriate horseradish peroxidase (HRP)-conjugated secondary antibodies for 1 h at 18 °C to 25 °C. Membranes were again washed eight times for 40 min, and proteins were visualized using West-Q Pico ECL solution (GenDEPOT, Katy, TX, USA) and detected using an Amersham ImageQuant 800 biomolecular imager (Cytiva, Marlborough, MA, USA). The signal intensities of immunoblot bands were quantified using ImageJ software (NIH, Bethesda, MD, USA) and Multi Gauge V3.0 software (Fujifilm, Tokyo, Japan). Primary and secondary antibodies were used for western blotting. JAK1 (D1T6W; 1:1000 dilution), p-JAK1(cat. no. 3331; 1:1000), JAK3 (5H2; 1:1000), p-JAK3 (D44E3; 1:1000), p-STAT5 (C11C5; 1:1000), AKT (cat. no. 9272; 1:1000), p-AKT (D9E; 1:1000), ERK1/2 (137F5; 1:1000), p-ERK1/2 (D13.14.4E; 1:2000), CIS (D4D9; 1:500) DUSP4 (D9A5; 1:500), SHP1 (C14H6; 1:1000), p-SHP1 (D11G5; 1:1000), SHP2 (D50F2; 1:1000), p-SHP2 (cat. no. 3703; 1:1000), SHIP1 (D1163; 1:500), p-SHIP1 (cat. no. 3941; 1:500), HRP-linked mouse IgG (cat. no. 7076; 1:2000), and HRP-linked rabbit IgG (cat. no. 7074; 1:2000) were purchased from Cell Signaling Technology (Danvers, MA, USA). DTX1 (cat. no. HPA055275; 1:500) was purchased from Atlas Antibodies (Bromma, Sweden), STAT5 (cat. no. 610191; 1:200) was purchased from BD Biosciences, and β-Actin (C4; 1:1000) was purchased from Santa Cruz Biotechnology (Dallas, TX, USA).

### Quantitative real-time PCR (qRT-PCT)

Total RNA was isolated using an RNeasy Micro kit (QIAGEN, Hilden, Germany) and converted to complementary DNA (cDNA) using an RT PreMix cDNA synthesis kit (iNtRON-Biotechnology, Seongnam, Republic of Korea), according to the manufacturer’s protocols. Quantitative real-time polymerase chain reaction (qPCR) was performed using a StepOne Plus or Viia7 system (Applied Biosystems) with qPCRBIO SyGreen Mix (PCR Biosystems, London, UK) according to the manufacturer’s instructions. Melting curve analysis was performed immediately after amplification to confirm primer specificity. Relative RNA expression was normalized to *Gapdh* messenger RNA (mRNA), a housekeeping gene, according to the 2^−△△Ct^ calculation method. The primers used were as follows:

*Cish*, 5′-GAACACACCAGCCACTGTCC-3′ (forward) and 5′-GCCAGCAAAGGACGAGGTC-3′ (reverse); *Socs1*, 5′-GTAGCACACAACCAGGTGGC-3′ (forward) and 5′-GGAGGAGGAAGAGGAGGAAGG-3′ (reverse); *Socs2*, 5′-TGCAAGGATAAGCGGACAGG-3′ (forward) and 5′-CTGCAGAGATGGTGCTGACG-3′ (reverse); *Socs3*, 5′-TTTCGCTTCGGGACTAGCTC-3′ (forward) and 5′-TTGCTGTGGGTGACCATGG-3′ (reverse); *Dtx1*, 5′-CCTGTGAATGGTCTGGGCTTC-3′ (forward) and 5′-CAGCGGCTGTGCTCATTCA-3′ (reverse); *Dusp4*, 5′-CTGGACTGCAGACCGTTCCT-3′ (forward) and 5′-GCCGCACGATGGTGTTACAG-3′ (reverse); *Ptpn14*, 5′-GCAAGAAAGGACGGTGTGGC-3′ (forward) and 5′-ACGGACCGACTGGATCTCCT-3′ (reverse); *Ptprf*, 5′-GCGCTTCGAGGTCATTGAGT-3′ (forward) and 5′-ATGGCTTCATCTCGCTGCAC-3′ (reverse); *Gapdh*, 5′-CAGCGACACCCACTCCTCCACCTT-3′ (forward) and 5′-CATGAGGTCCACCACCCTGTTGCT-3′ (reverse).

### Proteomic analysis

Mass analysis with three secretome sets from three independent donors was performed using the Proteomics Platform at ProteomTech Inc. (Seoul, Republic of Korea). Secretome sets were quantified using the Braford protein assay. In addition, 2-dimensional gel electrophoresis (2-DE) was performed for protein separation. Proteins were visualized using the Coomassie Brilliant Blue G-250 staining method. Computer analysis of the 2-DE images was performed using Image Master 2D Platinum Software (Cytiva). The expression level of each spot was determined by dividing the volume of each spot by the total volume of all the spots in the gel. After image analysis, spots were subjected to in-gel trypsin digestion and analyzed by liquid chromatography with tandem mass spectrometry (LC–MS/MS) using a nanoACQUITY UPLC and LTQ Orbitrap XL mass spectrometer (Thermo Fisher Scientific). Individual MS/MS spectra were processed using SEQUEST software (Thermo Fisher Scientific) and searched in the NCBI database using the MASCOT search program (Matrix Sciences, Chicago, IL, USA). Search parameters for data analysis were set as follows: carbamidomethyl (C) as a fixed modification, deamidated (NQ), and oxidation (M) as variable modifications, 10 ppm as tolerance of peptide mass, 0.8 Da as MS/MS ion mass tolerance, and 2 as allowance of missed cleavage. For bioinformatics analysis, GO terms were analyzed using the Database for Annotation, Visualization, and Integrated Discovery (DAVID) tools. Peptides were selected according to the significance threshold in the identity score of *P*-value ≤ 0.05.

### mRNA sequencing and analysis

Human primary NK cells purified from PBMCs of six healthy donors were used to identify the gene expression patterns of NK cells in the presence of the ADSC secretome. Total RNA was isolated using an RNeasy Micro kit (QIAGEN), according to the manufacturer’s protocols, and then sent to Macrogen Inc. (Seoul, Republic of Korea) for mRNA sequencing. The extracted RNA was checked for integrity using a 2100 Bioanalyzer instrument (Agilent Technologies, Santa Clara, Ca, USA) with an RNA Integrity Number (RIN) value. RNA libraries were prepared using a TruSeq stranded mRNA library kit (Illumina, San Diego, CA, USA), utilizing polyA selection of mRNA, according to the manufacturer’s protocols. Purified RNA was randomly fragmented for short-read sequencing, and cDNA was synthesized. After adding different adaptors to both ends of the synthesized cDNA fragments, they were amplified by PCR to an amount sufficient for sequencing, which was performed using a NovaSeq 6000 platform (Illumina). RNA-seq raw reads were used for quality control (QC) using the FastQC v0.11.7 program (Babraham Institute, Cambridge, UK) and then trimmed using Trimmomatic v0.38 to minimize errors that may occur during alignment. After trimming, RNA-seq reads were mapped to a population of human genomes using HISAT2 v2.1.0, and transcript assembly was performed using the StringTie v2.1.3b program to obtain the expression profile values. To verify differentially expressed genes (DEGs) between the two groups (control vs. treatment), the gene-level read counts obtained were normalized using the DESeq2 package, and 352 genes satisfying the conditions of |fc|≥ twofold pairwise change and nbinomWaldTest raw *P*-value < 0.05 were finally selected. These genes were grouped and visualized on a heatmap and dendrogram image by hierarchical clustering, volcano plot by the composite metric (log_2_fc ×  − log_10_ adjusted *P*-value), and dot plot by GO enrichment.

### Statistical analysis

Each experiment was independently repeated more than three times, and similar results were obtained. All statistical analyses were performed using GraphPad Prism 5 or 8 software (GraphPad Software, San Diego, CA, USA). Data are represented as mean ± SEM. Two-tailed Student’s *t*-tests for paired or unpaired data were used to determine the statistical significance between treatment and control values. In some experiments, data were analyzed using one-way analysis of variance (ANOVA) with Dunnett’s multiple comparisons test to compare the control group to all treatment groups. Statistical significance is denoted as follows: ns (not significant), *P < 0.05, **P < 0.01, and ***P < 0.001.

## Results

### The ADSC secretome does not affect NK-92 cells viability and proliferation

**Fig. 1:**
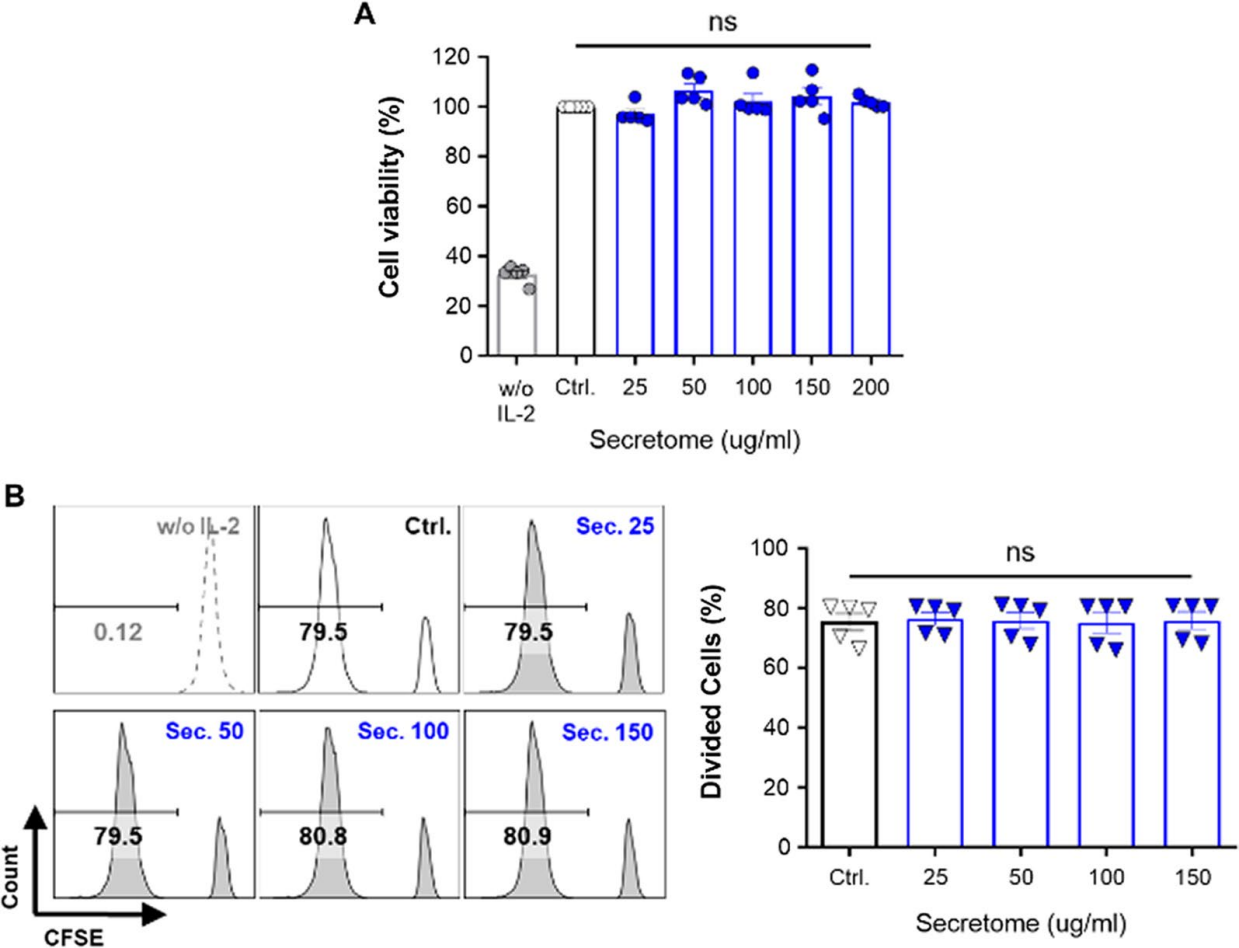
Intact viability and proliferation of NK-92 cells in the presence of the ADSC secretome. **A** Cell viability of IL-2-activated NK-92 cells incubated for 48 h with the ADSC secretome at various concentrations (horizontal axes) using CCK-8 assay. **B** Cell proliferation of IL-2-activated NK-92 cells treated as described in **A** for 96 h using CFSE staining assay. Error bars represent mean ± SEM of five independent experiments. Statistical significance was determined using one-way ANOVA with Dunnett’s multiple comparison test; not significant (ns)

As mentioned above, MSCs themselves have controversial effects on NK cells, but their conditioned media, named secretome, seem to inhibit the proliferation and activation of NK cells. Thus, we first investigated the effect of the ADSC secretome on the viability and proliferation of NK-92 cells, a human NK cell line, by adding the ADSC secretome at different concentrations from 25 to 200 μg/ml for 48 to 96 h. It is well known that IL-2 is essential to NK-92 cells for their survival and activation [44]. Therefore, IL-2 was used as a stimulus for NK cell activation in all experiments. No change was observed in cell viability at concentrations below 200 μg/ml of the ADSC secretome compared to the IL-2 alone control group (Fig. 1A). On the other hand, NK-92 cells rarely proliferated without IL-2 for 48 h. Moreover, we did not observe a substantial effect of the ADSC secretome on the proliferation of NK-92 cells, as assessed by CFSE dilution compared with the IL-2 control (Fig. 1B). Next, we examined the level of IFN-γ in the culture supernatant of NK-92 cells to determine the optimal inhibitory concentrations of the ADSC secretome. The ADSC secretome suppressed IFN-γ secretion by NK-92 cells at all concentrations at 48 h (Additional file 1: Figure S1). Hence, we carried out all experiments at non-toxic concentrations of the ADSC secretome, whose concentrations simultaneously inhibited cytokine secretion by NK-92 cells (25 and 150 μg/ml). Our results demonstrate that the ADSC secretome inhibits IFN-γ secretion by NK-92 cells but does not affect cell viability and proliferation.

### The ADSC secretome suppresses IL-2-induced activation of NK-92 cells

**Fig. 2:**
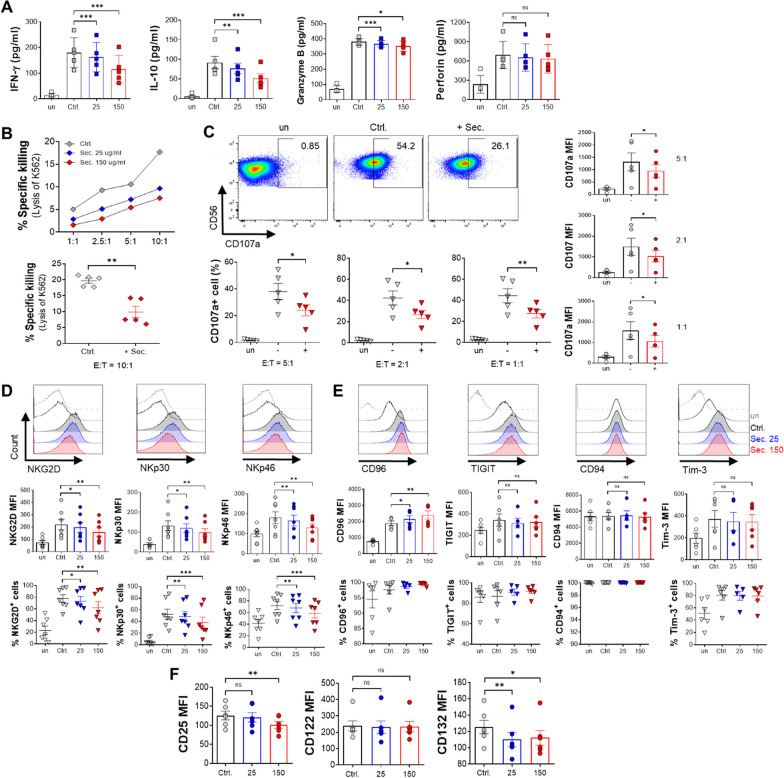
Alteration in effector function and receptor expression of NK cells by the ADSC secretome. NK-92 cells stimulated with IL-2 were cultured for 48 h with 25 (blue) or 150 μg/ml ADSC secretome (red), or without (gray). Unstimulated NK cells were also cultured for negative control (white). **A** The production of effector cytokines (IFN-γ and IL-10) and cytolytic granules (granzyme B and perforin) in the supernatant was measured by ELISA. **B** Cytotoxicity of NK-92 cells against K562 target cells at the indicated NK:K562 (E:T, effector:target) ratios of 1:1, 2.5:1, 5:1, or 10:1 (upper). Stimulated NK cells were treated with 150 μg/ml ADSC secretome for 48 h, followed by co-culture with K562 target cells at a 10:1 ratio for 4 h (bottom). **C** Degranulation marker from activated NK-92 cells was assessed using flow cytometry (CD56^+^ CD107a^+^) as shown dot blot (at a 5:1 ratio, upper), frequency of CD107a^+^ NK cells (at the indicated ratios, bottom), and the expression level of CD107a on NK cells (at the indicated ratios, right side). **D** to **F** Flow cytometry analysis of activating receptors, such as NKG2D, NKp30, and NKp46 (**D**; histogram, MFI, and frequencies), inhibitory receptors, such as CD96, TIGIT, CD94, and Tim-3 (**E**; histogram, MFI, and frequencies), and IL-2 receptors, such as CD25, CD122, and CD132 (**F**; MFI only). Error bars represent mean ± SEM of five to seven independent experiments. Statistical tests were determined by Student’s paired two-tailed *t*-test; not significant (ns), *P < 0.05, **P < 0.01, ***P < 0.001

Although many researchers have focused on the immunomodulatory ability of MSCs via the secretome over the past few years, it remains in disagreement with each other, especially for NK cells. To examine the immunomodulatory effect of the ADSC secretome on NK-92 cell activity, we initially measured the levels of effector cytokines or cytolytic granules in the supernatant of IL-2-stimulated NK cells, and then observed the cytolytic function and degranulation of NK cells, which presented granule exocytosis. High levels of IFN-γ, IL-10, and granzyme B, except for perforin, in the supernatant of IL-2-stimulated NK-92 cells were prevented by the ADSC secretome compared to that of the IL-2 alone control group in a dose-dependent manner (Fig. 2A). NK cell cytotoxicity toward K562 target cells, a human erythromyeloid leukemia cell line, was inversely dependent on the concentration of the ADSC secretome in an effector-to-target (E:T) ratio-dependent manner (Fig. 2B, upper). IL-2-activated NK cells treated with 150 μg/ml ADSC secretome exhibited a statistically significant reduction in their ability to kill target cells compared to IL-2 alone control NK cells (Fig. 2B, bottom). For the degranulation assay, abundant surface expression and frequency of CD107a^+^ NK cells were measured as functional markers. Unlike IL-2 alone control NK cells, ADSC secretome-treated NK cells also exhibited a substantially lower ability to release cytolytic granules, as evidenced by the lower frequency of CD107a^+^ NK cells (Fig. 2C, upper and bottom) and mean fluorescence intensity (MFI) (Fig. 2C, right) at all E:T ratios.

NK cell activity is generally governed by the integration of signals from the activating and inhibitory receptors on their surface [4, 5, 10, 45]. The activating receptors of NK cells are specific for ligands expressed on stressed, viral infected, or tumorigenic cells [46]. In contrast, their inhibitory receptors recognize the presence of MHC Class I molecules, thereby integrating multilateral signals from receptors to prevent unexpected NK-mediated damage to healthy cells [46, 47]. Given the results showing that the ADSC secretome suppressed NK cell activity, we next identified the expression profiles of activating and inhibitory receptors in NK cells. With increasing ADSC secretome concentration, the expression level and activating receptor-positive frequency, including NKG2D, NKp30, and NKp46, in NK-92 cells gradually decreased compared to the IL-2 alone control group (Fig. 2D). This downregulation of the natural cytotoxicity receptor (NCR) family, such as NKp30 and NKp46, and natural killer group 2D (NKG2D) causes decreased levels of cytokine secretion and reduced cytolytic function of NK cells [47, 48]. Meanwhile, the surface expression of inhibitory receptors, such as T-cell immunoglobulin and ITIM domain (TIGIT), CD94, and T-cell immunoglobulin mucin-3 (Tim-3), on NK-92 cells was stable even in the presence of the ADSC secretome, but interestingly, only the expression of CD96 among them was increased in a dose-dependent manner compared with the control group (Fig. 2E, medium). In contrast, the proportion of inhibitory receptor-positive NK cells was barely affected by the ADSC secretome, with very high rates in all the groups (Fig. 2E, bottom).

NK cell activity is governed by the integration of signals from cytokines such as IL-2 [9, 49]. IL-2 regulates immune responses by binding to three subunits of the IL-2 receptor (IL-2R), IL-2Rα (CD25), IL-2Rβ (CD122), and IL-2Rγ (CD132), with different affinities for IL-2 [50, 51]. Therefore, we tested the expression levels of the three IL-2R subunits to confirm the effect of the ADSC secretome on IL-2R in NK-92 cells. The ADSC secretome decreased CD25 and CD132, but the level of CD122 was comparable to that of IL-2 alone in NK-92 cells (Fig. 2F).

Together, these data confirm that the ADSC secretome constrains IL-2-mediated effector function of NK cells, including the secretion of effector cytokines and cytolytic granules, cytotoxicity, and degranulation. Furthermore, the mean expression level and the proportion of activating receptor-positive (NKG2D, NKp30, and NKp46) NK cells gradually decreased in accordance with the dose of ADSC secretome, whereas, among all inhibitory receptors, only CD96 expression increased with a high dose of secretome. Moreover, the ADSC secretome reduced the expression levels of IL-2Rα and IL-2Rγ in NK cells.

### The ADSC secretome targets JAK-STAT and AKT pathway via regulator CIS

**Fig. 3:**
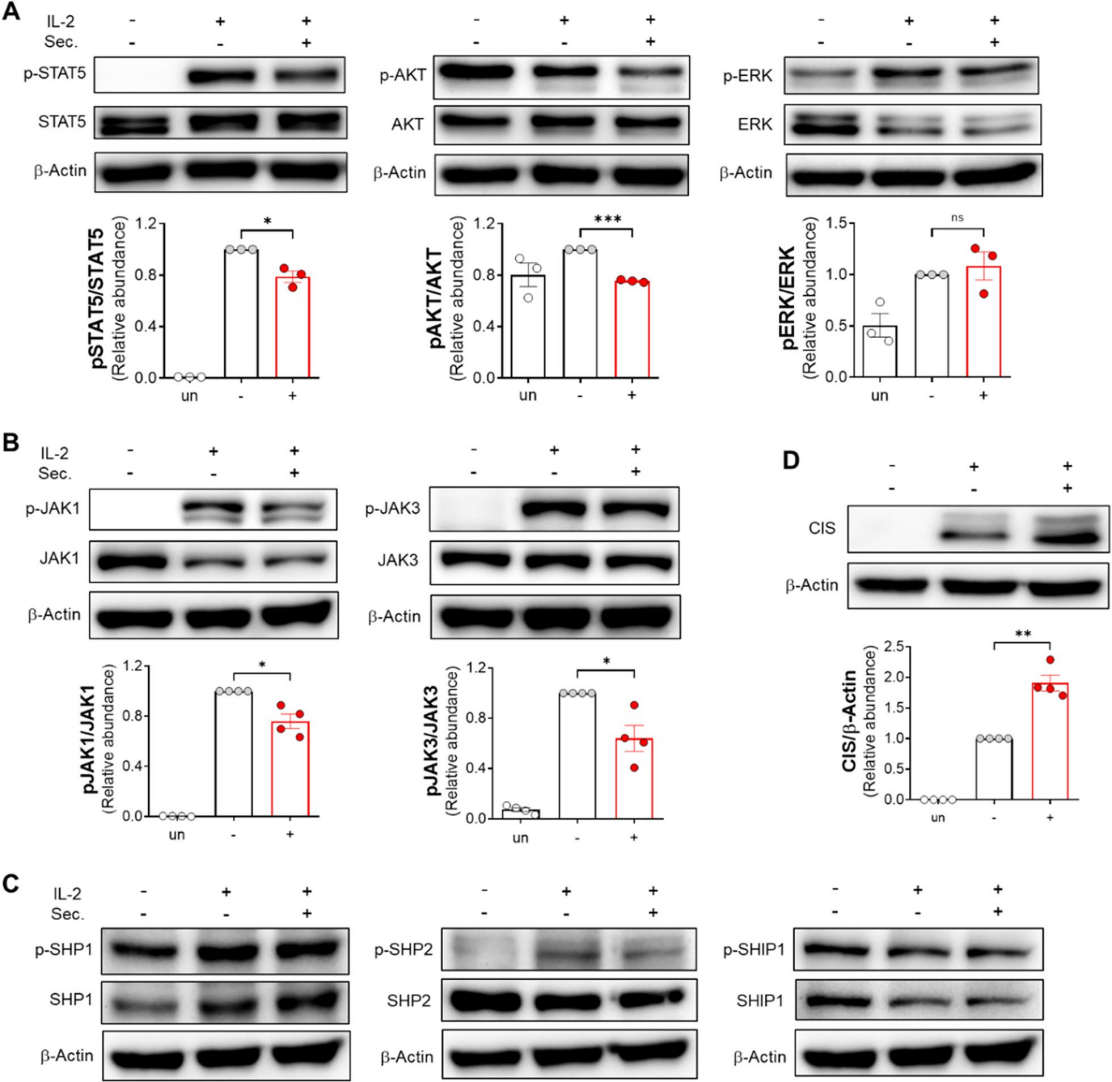
The ADSC secretome suppresses JAK-STAT and AKT signaling pathway by upregulation of regulator CIS. IL-2-stimulated NK cells were treated with or without 150 μg/ml ADSC secretome for 36 h. Unstimulated NK cells were also cultured for negative control. Immunoblot analysis of **A** STAT5, AKT, ERK, **B** JAK1, JAK3, **C** SHP1, SHP2, SHIP1, and **D** CIS expressions in NK-92 cells. Full-length blots were presented in Additional file 5: Figure S3 to S6. In the groups **A**, **B**, and **C**, the p- prefix denotes a phosphorylated protein. Error bars represent mean ± SEM of three to four independent experiments. Statistical tests were determined by Student’s paired two-tailed *t*-test; not significant (ns), *P < 0.05, **P < 0.01, ***P < 0.001

The cytokine IL-2 provokes three major signaling pathways that induce maturation, homeostasis, and activation: the JAK/STAT, phosphoinositide 3-kinase (PI3K)/protein kinase B (AKT), and extracellular signal-regulated kinase (ERK) 1/2 pathways [50, 52]. To investigate the mechanisms underlying the suppression of IL-2-mediated NK cell activity by the ADSC secretome, we initially analyzed the change in expression levels of phosphorylated proteins such as pSTAT5, pAKT, and pERK, the main downstream molecules of three major signaling pathways triggered by IL-2. Compared to the IL-2 alone control group, substantially reduced phosphorylation of STAT5 and AKT, but not ERK, was observed in IL-2-stimulated NK-92 cells upon treatment with the ADSC secretome (Fig. 3A; full-length blots were presented in Additional file 5: Figure S3). Next, we evaluated whether a considerable reduction in these downstream molecules is dependent on inactivation of IL-2Rβ/γ_c_-associated JAK1 and JAK3 tyrosine kinases. Addition of the ADSC secretome significantly reduced IL-2-supported phosphorylation of JAK1 and JAK3 without changes in total JAKs levels compared with IL-2 alone in NK-92 cells (Fig. 3B; full-length blots were presented in Additional file 5: Figure S4). This finding suggests that the ADSC secretome reduces JAK1 and JAK3 phosphorylation, thereby interfering with JAK/STAT and PI3K/AKT pathways by targeting pSTAT5 and pAKT.

We therefore asked which molecular factors could inhibit IL-2-mediated JAK activity in the NK cell cytoplasm. As previously reported, various negative regulators control JAK-STAT signaling through distinct mechanisms, including PTPs and SOCS proteins [11, 12]. These PTPs also play a crucial role in inhibitory receptor-mediated suppression by inducing the dephosphorylation of signaling proteins linked to activating receptors in NK cells [5, 10, 53]. Meanwhile, the SOCS family is induced by many cytokines to negatively regulate cytokine signaling via inhibition of JAT/STAT activation and induction of proteasomal degradation of signaling substrates [11, 12]. First, we investigated which cellular phosphatases (SHP-1, SHP-2, and SHIP-1) are required for JAK/STAT inhibition in NK-92 cells. Despite a significant decrease in the phosphorylation of JAK1 and STAT5 by treatment of NK-92 cells with the ADSC secretome, we did not observe any increase in the levels of these phosphatases SHPs or SHIP (Fig. 3C; full-length blots were presented in Additional file 5: Figure S5). The significant reduction in JAK1, JAK3, and STAT5 phosphorylation by the ADSC secretome in NK cells was not due to these phosphatase levels. However, protein CIS among the SOCS family was upregulated during IL-2-mediated activation, and interestingly, these increases were more prominent upon treatment with the ADSC secretome (Fig. 3D; full-length blots were presented in Additional file 5: Figure S6), indicating that protein CIS among the SOCS family may be a major regulator of NK cell activity through the ADSC secretome.

### The ADSC secretome also constrains effector function of human primary NK cells

**Fig. 4:**
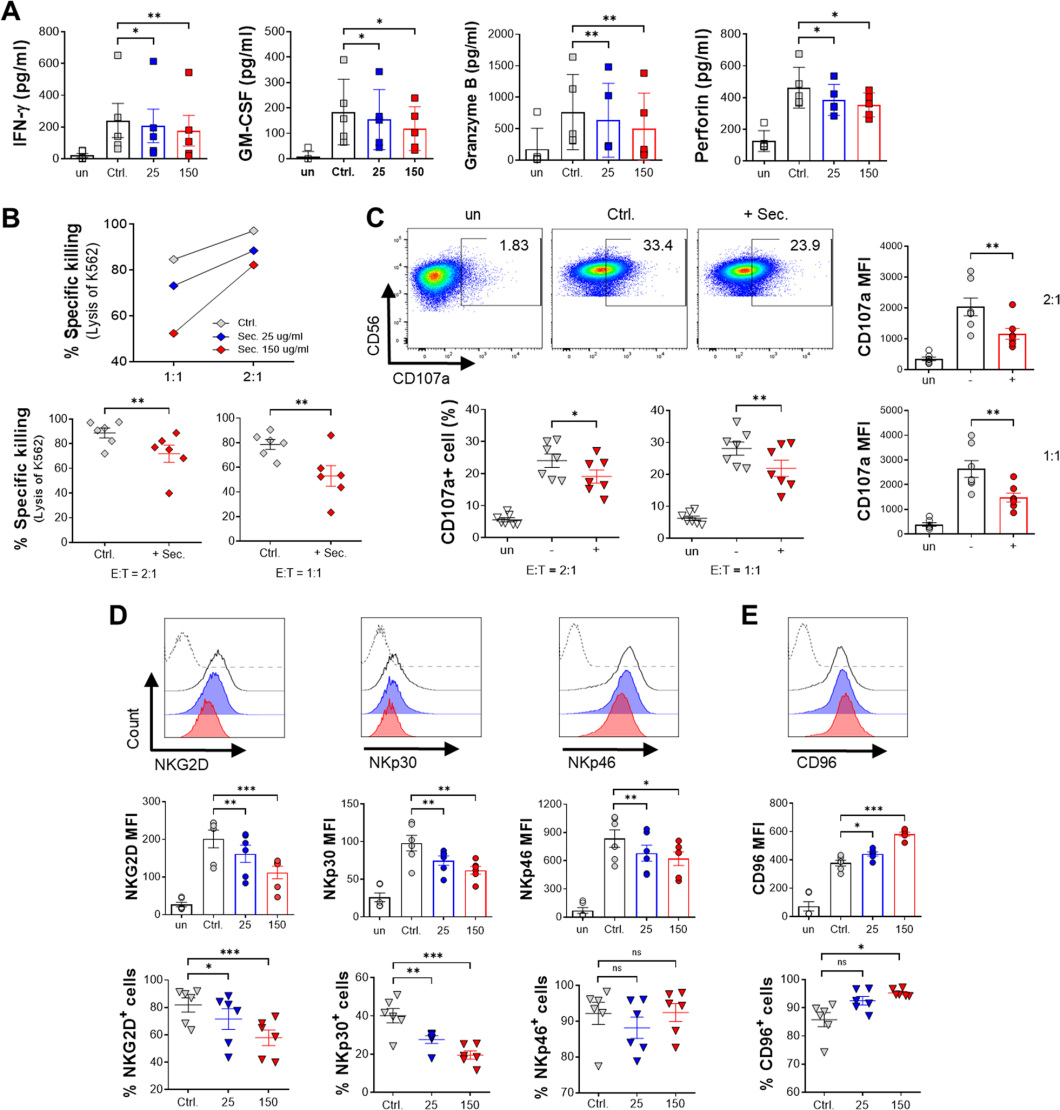
Alteration in effector function and receptor expression of human primary NK cells by the ADSC secretome. Human primary NK cells isolated from PBMCs of a healthy donor were cultured for 48 h with 25 (blue) or 150 μg/ml ADSC secretome (red), or without (gray) in the presence of 10 ng/ml of rhIL-2. Unstimulated NK cells were also cultured for negative control (white). **A** The production of effector cytokines (IFN-γ and GM-CSF) and cytolytic granules (granzyme B and perforin) in the supernatant was measured by ELISA. **B** Cytotoxicity of primary NK cells against K562 target cells at the indicated NK:K562 (E:T, effector:target) ratios of 1:1, or 2:1 (upper). Stimulated primary NK cells were treated with 150 μg/ml ADSC secretome for 48 h, followed by co-culture with K562 target cells at the indicated ratios for 4 h (bottom). **C** Degranulation marker from activated primary NK cells was assessed using flow cytometry (CD3^−^ CD56^+^ CD107a^+^) as shown dot blot (at a 1:1 ratio, upper), frequency of CD107a^+^ NK cells (at the indicated ratios, bottom), and the expression level of CD107a on primary NK cells (at the indicated ratios, right side). **D** to **E** Flow cytometry analysis of activating receptors, such as NKG2D, NKp30, and NKp46 (D; histogram, MFI, and frequencies), inhibitory receptor CD96 (E; histogram, MFI, and frequencies). Error bars represent mean ± SEM of five to seven healthy donors. Statistical tests were determined by Student’s paired two-tailed *t*-test; not significant (ns), *P < 0.05, **P < 0.01, ***P < 0.001

To confirm the ADSC secretome-mediated suppression of the effector function of human primary NK cells as observed in the NK-92 cell line, whole blood samples were collected from healthy donors, and NK cells were isolated from peripheral blood mononuclear cells (PBMCs). We also examined the production of effector cytokines and cytolytic granules by primary NK cells in the presence or absence of the ADSC secretome. In agreement with the above experiments using NK-92 cells, the ADSC secretome also inhibited the secretion of effector molecules of human primary NK cells in a concentration-dependent manner, as demonstrated by the decreased production of IFN-γ, GM-CSF, granzyme B, and perforin (Fig. 4A). Meanwhile, unstimulated NK cells lacking IL-2 secrete a few cytokines. Furthermore, when we examined their ability to kill target cells, we observed significantly decreased cytotoxic activities of human primary NK cells in the presence of the ADSC secretome compared with IL-2 alone control NK cells, consistent with our finding in the NK-92 cell line (Fig. 4B). Moreover, the degranulation assay further proved that primary NK cells cultured with the ADSC secretome also exhibited a significant decrease in the frequency and MFI of membrane CD107a against K562 cells compared with control NK cells (Fig. 4C). These results indicate that the inhibitory efficacy of the ADSC secretome is sufficient for human primary NK cells.

Next, the expression patterns of NK cell activating and inhibitory receptors were measured using flow cytometry. We found that, like NK-92 cells, human primary NK cells administered the ADSC secretome had markedly decreased expression levels and percentages of activating receptors, except for the proportion of NKp46^+^ cells (Fig. 4D), while they had an increased expression level and percentage of inhibitory receptor CD96 compared with the control group (Fig. 4E). These data indicate that the result of human primary NK cells is consistent with the result of NK-92 cells that the ADSC secretome has a dose-dependent effect on decreasing expression levels of NKG2D, NKp30, and NKp46, while increasing the expression level of CD96 in NK-92 cells.

### Proteomic profiling of the ADSC secretome identifies the putative factors for immunomodulation

To discern the protein composition released by ADSCs, the proteome of the ADSC secretome was analyzed by LC–MS/MS with a combination of 2-DE gel electrophoresis and gel digestion. The three independent ADSC secretome samples from individual donors were separated from 15 to 250 kDa in the pH range of 3 to 10. After comparing protein patterns among the three samples, the secretome sample of donor 1, which shows the most remarkable suppression effect, was finally selected and found to have 71 protein spots (Additional file 1: Figure S2A and Additional file 2: Table S1). Through LC–MS/MS profiling and bioinformatics analysis, we found that 83 proteins were included in the ADSC secretome, based on the criteria for selecting spots with a ratio value of twofold or less in the two 2-DE images (Additional file 3: Table S2). Most of these results were consistent with the proteins detected in previous studies.

To establish the functional significance of the identified 83 proteins within the ADSC secretome, gene ontology (GO) was performed for enrichment analysis using gene annotations that afforded a structuralized model for individual genes in accordance with the biological process (BP), molecular function (MF), and cellular component (CC) of the gene (Additional file 1: Figure S2B). First, in terms of BP, the largest proportion (20.7%) of the 83 proteins we found was related to extracellular matrix organization, and only 7.3% were involved in the innate immune response. In addition, they were also in categories related to the negative regulation of endopeptidase activity (11%), proteolysis (8.5%), cellular response to amino acid stimulus (7.3%), and cellular protein metabolic process (6.1%). In terms of MF, the largest proportion (61%) of 83 proteins we found was related to protein binding and the next largest was in categories related to calcium ion binding (18.3%). Lastly, in terms of CC, the largest proportion (74.4%) of 83 proteins was related to extracellular exosomes, which play a pivotal role in the immunomodulatory effects of MSCs. These results suggest that the ADSC secretome may affect immune regulation via proteins involved in these cellular processes.

The ADSC secretome exhibited consistent effects on both types of NK cells representing humans, such as the cell line NK-92 and primary NK obtained from blood as shown above, but the soluble protein components within the ADSC secretome that may cause immune suppressive effects need to be revealed clearly. Therefore, we next performed a functional enrichment analysis of the whole data set and selected the list of 12 proteins with immunomodulatory potential within the ADSC secretome (Additional file 1: Figure S2C), particularly those more related to NK cells, through the investigation and integration of earlier studies on MSC’s proteins. These proteins possess multiple biological functions, including structural composition, cell migration, adhesion, proliferation, development, and immune regulation.

**Fig. 5:**
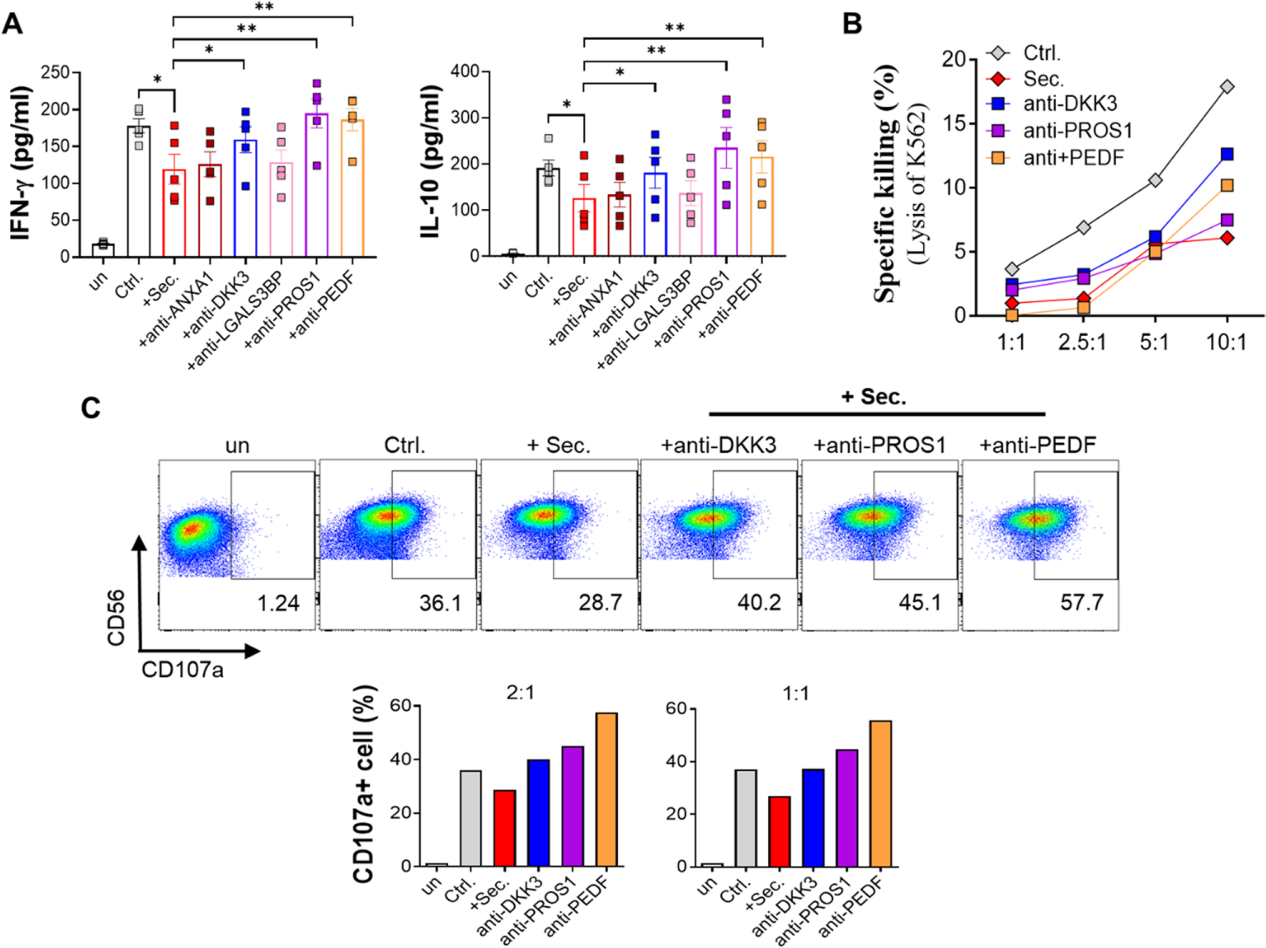
Efficacy of the ADSC secretome to effector function of NK-92 cells when each immunomodulatory candidate within the ADSC secretome was blocked by neutralizing antibody. **A** to **C** NK-92 cells stimulated with IL-2 (5 ng/ml) were incubated for 48 h with the ADSC secretome (150 μg/ml) and neutralizing antibodies (1 μg/ml) to each candidate to examine recovery of cellular activity. Unstimulated NK cells were cultured for negative control. **A** The concentrations of IFN-γ and IL-10 in the culture supernatants was measured by ELISA. Error bars represent mean ± SEM of five independent experiments. Statistical tests were determined by Student’s paired two-tailed t-test; *P < 0.05. Anti-ANXA1, anti-annexin A1 antibody; anti-LGALS3BP, anti-galectin-3-binding protein antibody. **B** After incubation, NK-92 cells were co-cultured with K562 target cells for 4 h at the indicated E:T ratios, followed by measurement of cytotoxicity of NK-92 cells against K562 cells. **C** NK-92 cells were stained with PE-conjugated CD107a antibodies and co-cultured with K562 cells for 4 h at the indicated E:T ratios to measure the level of exocytosis of NK cells toward target cells. Degranulation marker CD107a was assessed using flow cytometry as shown dot blot (left) and frequency of CD107a^+^ NK cells (right). Representative data were shown

Therefore, we investigated whether the suppressed effector function of NK cells via the ADSC secretome could recover when we used a neutralizing antibody against each immunomodulatory candidate of the ADSC secretome. Interestingly, five neutralizing antibodies out of 12 restored the activities of NK cells exposed to the ADSC secretome, and anti-dickkopf-related protein 3 (DKK3), anti-pigment epithelium-derived factor (PEDF), and anti-protein S (PROS1) showed remarkable recovery (Fig. 5A-C). Our results suggest that the inhibitory effect of the ADSC secretome on NK cell activity may be partially dependent on these novel candidates and the previously known major regulatory proteins.

### Activated NK cells present substantial changes along with DUSP4 in response to the ADSC secretome

**Fig. 6:**
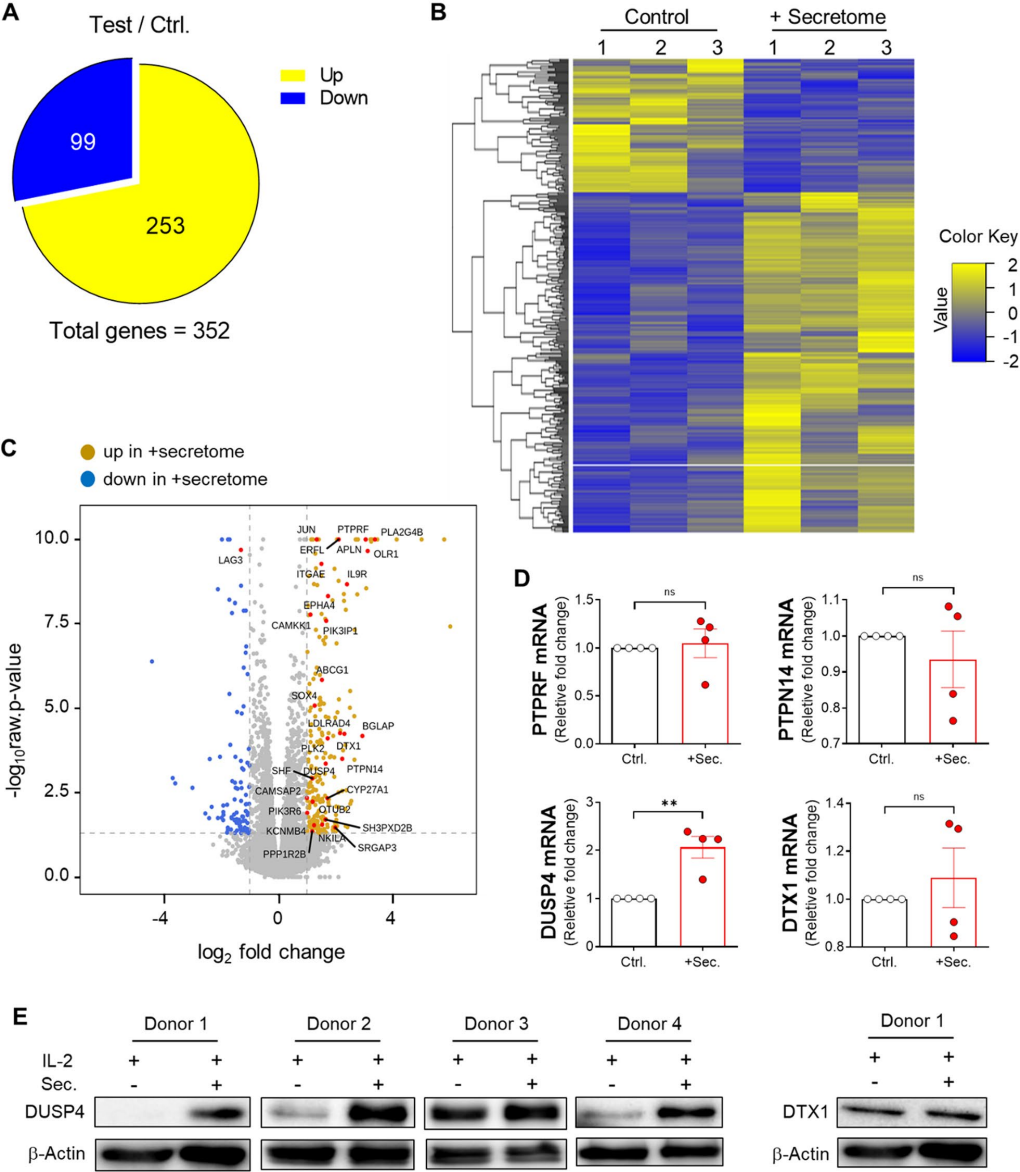
Human NK cells showed transcriptome changes that include the expression of cell activity– regulated genes and upregulation of DUSP4 expression caused by the ADSC secretome. Human primary NK cells isolated from PBMCs of a healthy donor were cultured for 48 h with 150 μg/ml of the ADSC secretome or without in the presence of 10 ng/ml of rhIL-2 (*n* = 6). **A** A total of 352 differentially expressed genes (DEGs) were indicated into 99 down-regulated genes (blue) and 253 up-regulated genes (yellow) in secretome-treatment group compared to IL-2 alone control group based on fold change and *P* value (*P* < 0.05, and |fc|≥ 2). **B** 352 DEGs indicated in **A** were grouped by the similarity of expression level and visualized in the heat map of the one-way Hierarchical Clustering using Z-score for normalized value (log_2_ based), ranging from low expression (blue) to high expression (yellow). **C** Volcano plot of gene expression of human primary NK cells using mRNA-seq (*n* = 6), indicating selected genes (red) associated with regulation of cell activity. Down-regulated (blue) and up-regulated (mustard) differentially expressed genes in secretome-treatment group were indicated. **D** mRNA levels of up-regulated differentially expressed genes that are phosphatase or ubiquitin ligase among the genes displayed in **C**. **E** Immunoblot analysis of protein levels of DUSP4 (left) and DTX1 (right) in NK cells from human blood. Full-length blots were presented in Additional file 5: Figure S7. RNA-seq data satisfying the conditions of adjusted *P* < 0.05, and |fc|≥ 2 were shown. Error bars represent mean ± SEM of four healthy donors. Statistical tests were determined by Student’s paired two-tailed *t*-test; not significant (ns), **P < 0.01

Next, we sought to elucidate the molecular mechanisms that drive the suppression of the IL-2-mediated activity of NK cells by the ADSC secretome. Messenger RNA sequencing (mRNA-seq) for transcriptional expression profiling was performed using human blood NK cells from six healthy donors. Interestingly, there was a significant difference in gene expression following ADSC secretome treatment for 48 h compared to that in the IL-2 alone control group. Among the 352 differentially expressed genes (DEGs) having more than two-fold change, 253 were upregulated, whereas 99 were downregulated (Fig. 6A and Additional file 4: Table S3). To arrange samples and genes with similar expression patterns, we conducted hierarchical clustering analysis of all the significant DEGs based on normalized values for each gene in each sample (Fig. 6B). We then examined the GO classifications of all 352 genes, which were allocated to the corresponding GO terms. We found that IL-2-activated NK cells treated with the ADSC secretome showed relatively high expression of several genes associated with phosphatase and ubiquitin ligase subunits, including *ptprf*, *ptpn14*, *dusp4*, and *dtx1* (Fig. 6C), which may contribute to the decreased levels of phosphorylated JAK1 and JAK3. We then performed real-time PCR to confirm whether these genes are indeed highly expressed in human blood NK cells treated with the ADSC secretome and found that only *dusp4* gene was notably upregulated compared with the IL-2 alone control group (Fig. 6D). Moreover, an increased expression level of DUSP4 protein was observed, unlike protein deltex-1 (DTX1), with no change in the ADSC secretome-treated group (Fig. 6E; full-length blots were presented in Additional file 5: Figure S7). Thus, these results indicate that the ADSC secretome results in increased expression of the mRNA and protein of phosphatase DUSP4 in IL-2-activated NK cells.

## Discussion

In this study, we aimed to provide invaluable insights into the basic biology of the regulation of NK cell activity by the MSC secretome. We closely examined the expression pattern of surface receptors of NK cells, identified negative regulators, and elucidated modulated signaling molecules for NK cell activity. Additionally, we presented novel candidates for immune regulators of MSCs. Our findings contribute to the understanding of NK cell regulation by the MSC secretome and suggest its potential as a therapeutic tool for treating NK-mediated inflammatory and autoimmune diseases.

Previous studies on the regulation of NK cell activity by the MSC secretome [39, 40, 42, 43] have focused on specific aspects, resulting in fragmentary information. However, our study aimed to provide a comprehensive understanding of the interaction between NK cells and the MSC secretome. We compared our findings to the existing literature and observed some discrepancies in the effects of the MSC secretome on NK cell functions. These inconsistencies highlight the need for further research to unveil the precise effects of the MSC secretome on NK cells.

We observed that the ADSC secretome specifically impacted the effector function of NK cells, such as cytokine secretion (i.e., IFN-γ, IL-10, granzyme B, GM-CSF, and perforin), cytotoxicity, and degranulation, while not affecting their proliferation. We identified the upregulation of the inhibitory receptor CD96 and the downregulation of activating receptors (i.e., NKG2D, NKp30, and NKp46) as key factors contributing to this effect. Despite the well-known inhibitory role of CD96, its precise function in NK cells remains elusive [54–57]. Our study suggests that CD96 might be involved in constraining NK cell activation rather than supporting it.

Additionally, we observed reduced expression levels of IL-2Rα and IL-2Rγ in NK cells exposed to the ADSC secretome. This finding suggests that the ADSC secretome may contribute to the decreased responsiveness of NK cells to IL-2 by obstructing the interaction of IL-2 with the IL-2Rαβγ trimeric complex, which possesses the greatest binding affinity to its ligand IL-2 [51]. However, the mechanisms underlying the regulation of IL-2R subunit expression by the MSC secretome require further investigation.

Our study also shed light on the signaling pathways affected by the ADSC secretome in NK cells. We observed a decreased magnitude of IL-2-stimulated JAK1 and JAK3 phosphorylation, leading to attenuated STAT5 and AKT phosphorylation. These findings indicate that the ADSC secretome dampens IL-2 signaling in NK cells without suppressing cell proliferation. It is likely that the PI3K/AKT pathway, crucial for NK cell effector function [58–60], is diminished by the ADSC secretome, while a minimum essential isoform of pSTAT5 maintains STAT5-driven survival signals [61].

Here, we demonstrated that during the inhibition of NK cell function by the ADSC secretome, CIS, but not SHPs or SHIPs, was upregulated in the cytoplasm, leading to the degradation or dephosphorylation of pJAK1, pJAK3, and pSTAT5 [11, 12, 62, 63]. This finding is supported by a study on a negative regulator in NK cells, which indicated that CIS is a critical suppressor of IL-15 signaling in NK cells via interaction with the activation loop peptides of JAK1 and JAK3 [7]. Interestingly, we also observed upregulation of DUSP4 by the ADSC secretome in human primary NK cells isolated from peripheral blood using mRNA sequencing, qRT-PCR, and western blotting. Phosphatase DUSP4, the major DUSP expressed in some immune cells, including NK cells, regulates MAP kinase activity to alter the inflammatory response induced by LPS [64] and STAT5 activity for T cell differentiation [65]. Although the findings reported here may not fully reflect the direct relationship between CIS and DUSP4 and the negative regulation of IL-2 signaling in NK cells, their upregulation may be a noteworthy observation in an effort to regulate inappropriate or excessive activation of NK cells.

Our results highlight the immunomodulatory effect of soluble factors of MSCs on NK cells activated by IL-2 and its regulatory mechanisms. The MSC secretome comprises various molecules with immunomodulatory potential. Previous studies have identified several proteins within the MSC secretome that affect NK cell activity. In our study, we characterized the contents of the ADSC secretome through proteomic analysis and identified 12 immunomodulatory factors. Moreover, we verified their immunomodulatory potential using neutralizing antibody against each candidate of the ADSC secretome.

The modulation of IL-2-mediated activation of NK cells through downstream signaling molecules and inhibitory receptors using the ADSC secretome holds therapeutic potential for altering NK cell function in autoimmune conditions. By elucidating the regulatory mechanisms and identifying key negative regulators of NK cell activity via the MSC secretome, we aim to pave the way for the development of promising biological agents and MSC secretome-based immunoregulatory therapies. Future research should focus on unraveling the precise mechanisms involved and exploring the therapeutic potential of the MSC secretome in immune-mediated disorders.

## Conclusions

**Fig. 7:**
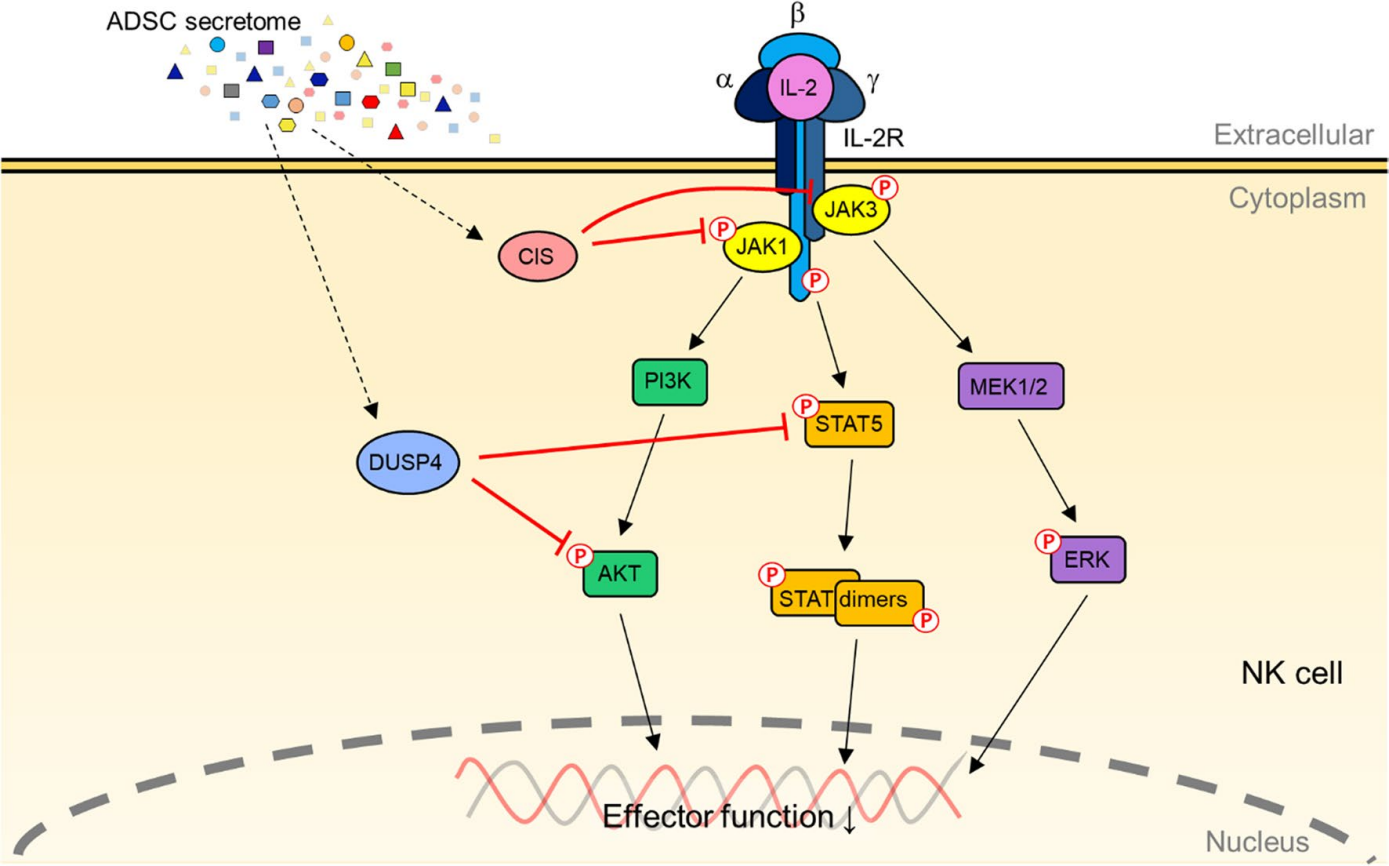
Possible mechanism of the regulation of IL-2 signaling pathway via CIS and DUSP4 increased by the ADSC secretome. The ADSC secretome induces upregulation of CIS and DUSP4 in NK cells, leading to attenuated effector function of NK cells while maintaining cell proliferation. CIS binds to pJAK1 and pJAK3 and targets them to proteasomal degradation to suppress their enzymatic activity. DUSP4 dephosphorylates pSTAT5 and pAKT to weaken the amplitude of IL-2 signaling cascade

In summary, we focused on the effects and detailed mechanism of soluble factors obtained from ADSCs culture media, named ADSC secretome, on NK cells, and not on the interaction between both cells. We have shown that the ADSC secretome restricted IL-2-mediated effector function of NK cells while maintaining proliferative potency, accompanied by elevated surface expression of NK inhibitory receptor CD96 and decreased surface expression of NK activating receptors, as well as lower expression of alpha chain CD25 and gamma chain CD132 of the IL-2 receptor. As a consequence, in the presence of the ADSC secretome, NK cells activated by IL-2 possessed a weakened IL-2 signaling by suppressing the phosphorylation of JAK1 and JAK3, while the increased induction of CIS and DUSP4 (Fig. 7). To demonstrate the ability of the ADSC secretome to regulate NK cell activity, we used secretome proteomic analysis and narrowed down the list of 12 candidates associated with immunomodulation of the ADSC secretome with the exception of that of previously known MSCs. Our findings provide a basis for determining the therapeutic potential of regulating NK cell function via the ADSC secretome in inflammatory and autoimmune diseases, characterized by inappropriate or excessive activation of NK cells, as well as the fundamental principle of secretome operation on NK cells.

### Supplementary Information

**Additional file 1: Fig. S1.** Screening of NK-inhibitory concentrations of the ADSC secretome. **Fig. S2**. Proteomic and gene ontology (GO) analysis of the ADSC secretome and identification of immunomodulatory candidates within the ADSC secretome.

**Additional file 2: Table S1.** Complete list of secreted proteins of ADSCs identified from 71 protein spots.

**Additional file 3: Table S2.** 83 proteins within the ADSC secretome.

**Additional file 4: Table S3.** 352 differentially expressed genes (DEGs) in secretome-treatment group.

**Additional file 5: Fig. S3-S7.** Uncropped full-length blots of western blot images.

## Data Availability

The mass spectrometry proteomics data have been deposited to the ProteomeXchange Consortium via the iProX partner repository with the dataset identifier PXD045254. The sequencing data have been deposited in NCBI’s Gene Expression Omnibus (GEO) and are publicly available under [accession number: GSE237253]. All other data including proteomics dataset generated from LC–MS/MS and/or analyzed during the current study are included within the article and its Supplementary Information files or available from the corresponding author upon reasonable request.

## Abbreviations

ADSC: Adipose tissue-derived stem cell
AKT: Protein kinase B (or PKB)
ANXA1: Annexin A1
BMSC: Bone marrow-derived stem cell
BP: Biological process
CC: Cellular component
CCK-8: Cell counting kit 8
CCL: Chemokine (C–C motif) ligand
CD: Cluster of differentiation
CFSE: Carboxyfluorescein diacetate succinimidyl ester
CIS: Cytokine-inducible Src homology 2 (SH2)-containing protein
COL1A1: Collagen alpha-1(I) chain
COL3A1: Collagen alpha-1(III) chain
DAVID: Database for annotation, visualization, and integrated discovery
DEG: Differentially expressed genes
DKK-3: Dickkopf-related protein 3
DTX1: Protein deltex-1
DUSP4: Dual specificity phosphatase 4
E:T: Effector:Target
ELISA: Enzyme-linked immunosorbent assay
ERK: Extracellular signal-regulated kinase
FN1: Fibronectin
GM-CSF: Granulocyte–macrophage colony-stimulating factor
GO: Gene ontology
GSN: Gelsolin
IFN-γ: Interferon γ
IL: Interleukin
IL-2R: Interleukin 2 receptor
ITAM: Immunoreceptor tyrosine-based activation motifs
ITIM: Immunoreceptor tyrosine-based inhibition motifs
JAK: Janus kinase
LC–MS/MS: Liquid chromatography with tandem mass spectrometry
LGALS3BP: Galectin-3-binding protein
LTF: Lactotransferrin
MF: Molecular function
MFI: Mean fluorescent intensity
MHC: Major histocompatibility complex
mRNA-seq: Messenger RNA sequencing
MSC: Mesenchymal stem cell
MWCO: Molecular weight cut-off
NCR: Natural cytotoxicity receptor
NK: Natural killer
NKG2D: Natural killer group 2D
qRT-PCR: Quantitative real-time polymerase chain reaction
PBMC: Peripheral blood mononuclear cell
PEDF: Pigment epithelium-derived factor
PI3K: Phosphoinositide 3-kinase
PROS1: Vitamin K-dependent protein S
PTP: Protein tyrosine phosphatases
PTPN14: Tyrosine-protein phosphatase non-receptor type 14
PTPRF: Receptor-type tyrosine-protein phosphatase F
SDS-PAGE: Sodium dodecyl sulfate polyacrylamide gel electrophoresis
SHIP: Src homology 2 (SH2) domain-containing inositol phosphatase
SHP: Src homology 2 (SH2) domain-containing protein tyrosine phosphatase
SOCS: Suppressor of cytokine signaling
SPARC: Secreted protein acidic and rich in cysteine
STAT: Signal transducer and activator of transcription
TFF: Tangential flow filtration
TIGIT: T-cell immunoglobulin and ITIM domain
Tim-3: T-cell immunoglobulin mucin-3
TNF-α: Tumor necrosis factor α
VTN: Vitronectin
