# Supplementary material for: ADSC secretome constrains NK cell activity by attenuating IL-2-mediated JAK-STAT and AKT signaling pathway via upregulation of CIS and DUSP4
Source: Stem Cell Res Ther. 2023 Nov 14;14:329. doi: 10.1186/s13287-023-03516-z (PMC10648656; doi:10.1186/s13287-023-03516-z)
Supplement: Supplementary file 1 — Additional file 1: Fig. S1. Screening of NK-inhibitory concentrations of the ADSC secretome. Fig. S2. Proteomic and gene ontology (GO) analysis of the ADSC secretome and identification of immunomodulatory candidates within the ADSC secretome. [file 13287_2023_3516_MOESM1_ESM.docx]

**Supplementary Figures**

**Figure S1**

**Screening of NK-inhibitory concentrations of the ADSC secretome.** The level of IFN-γ secretion of NK-92 cells treated with the ADSC secretome at various concentrations (horizontal axis) after stimulation with 5 ng/ml of rhIL-2 for 48 h. IFN-γ concentration from the culture supernatant of NK-92 cells was estimated by ELISA. Data represent two or three independent experiments and error bars indicate mean $\pm$ SEM.

**Figure S2**

**
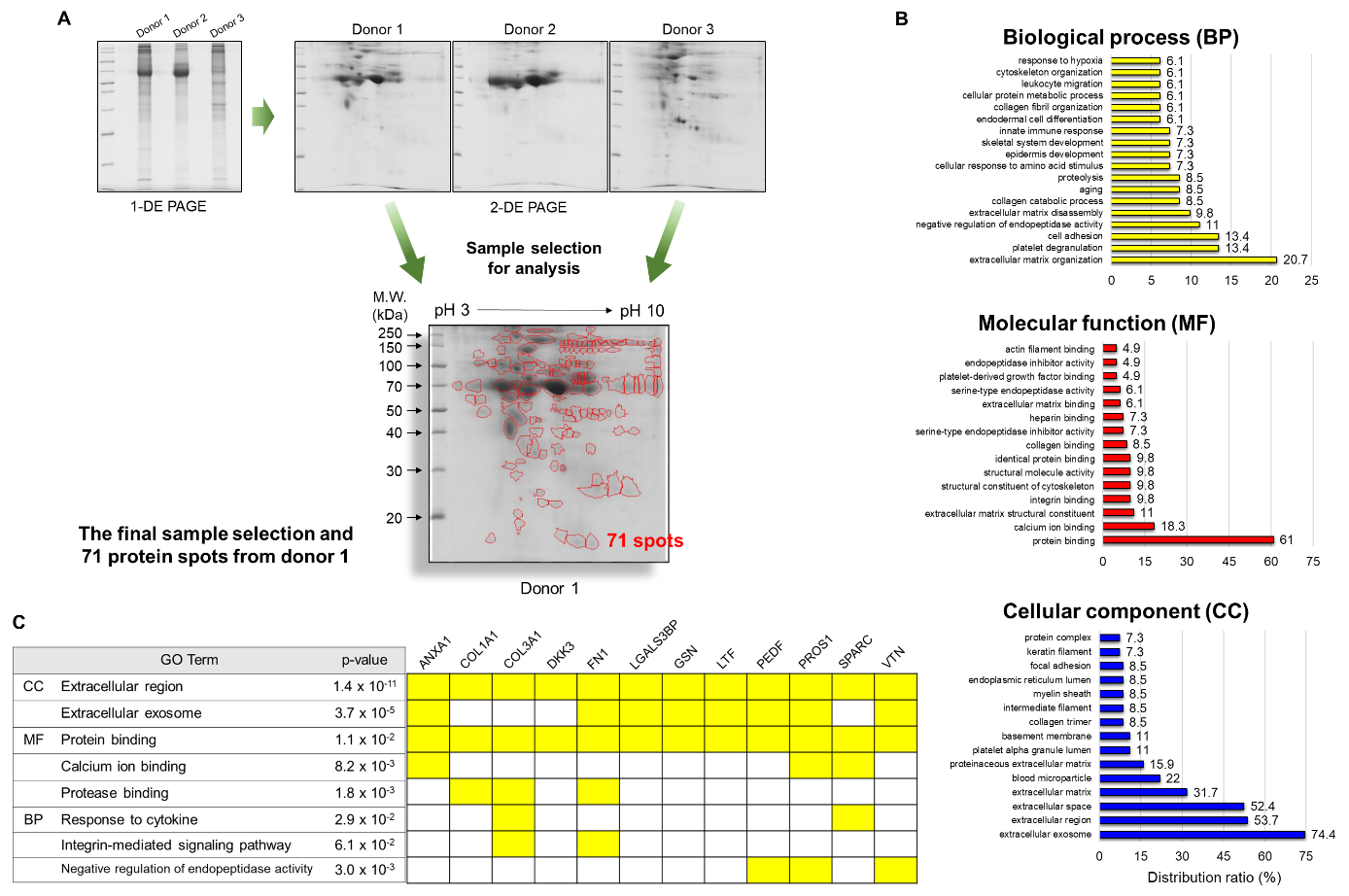
**

**Proteomic and gene ontology (GO) analysis of the ADSC secretome and identification of immunomodulatory candidates within the ADSC secretome.** (A) Workflow of secretome profiling before LC-MS/MS analysis. 1-DE and 2-DE gel electrophoresis were performed with ADSC secretome sets from 3 different donors to identify protein patterns and to fractionate proteins. Prior to LC-MS/MS analysis to identify protein composition, 71 protein spots were obtained from donor 1 upon 2-DE electrophoresis and finally developed using Image MasterTM 2D Platinum (GE Healthcare, Chicago, IL). (B) GO enrichment analysis of identified 83 proteins from the ADSC secretome was performed against the desired protein database using the algorithm of the DAVID tools. Categories with P value < 0.05 were listed for each type. (C) A list of immunomodulatory proteins (right) likely to be more relevant to NK cell biology. Each protein was arranged in the representative GO terms (left) of cellular compartments (CC), molecular functions (MF), and biological processes (BP) that were selected in order of highest protein content using the algorithm of the DAVID tools. ANXA1, annexin A1; COL1A1, collagen alpha-1(I) chain; COL3A1, collagen alpha-1(III) chain; DKK3, dickkopf-related protein 3; FN1, fibronectin; LGALS3BP, galectin-3-binding protein; GSN, gelsolin; LTF, lactotransferrin; PEDF, pigment epithelium-derived factor; PROS1, vitamin K-dependent protein S; SPARC, secreted protein acidic and rich in cysteine; VTN, vitronectin.
