# Supplementary material for: ADSC secretome constrains NK cell activity by attenuating IL-2-mediated JAK-STAT and AKT signaling pathway via upregulation of CIS and DUSP4
Source: Stem Cell Res Ther. 2023 Nov 14;14:329. doi: 10.1186/s13287-023-03516-z (PMC10648656; doi:10.1186/s13287-023-03516-z)
Supplement: Supplementary file 5 — Additional file 5: Fig. S3-S7. Uncropped full-length blots of western blot images. [file 13287_2023_3516_MOESM5_ESM.docx]

**Uncropped full-length blots**

**Figure S3. Full-length blots of western blot images in figure 3A.**

(A: p-STAT5; B: STAT5; C: β-Actin; D: p-ATK; E: AKT; F: β-Actin; G: p-ERK; H: ERK; I: β-Actin)


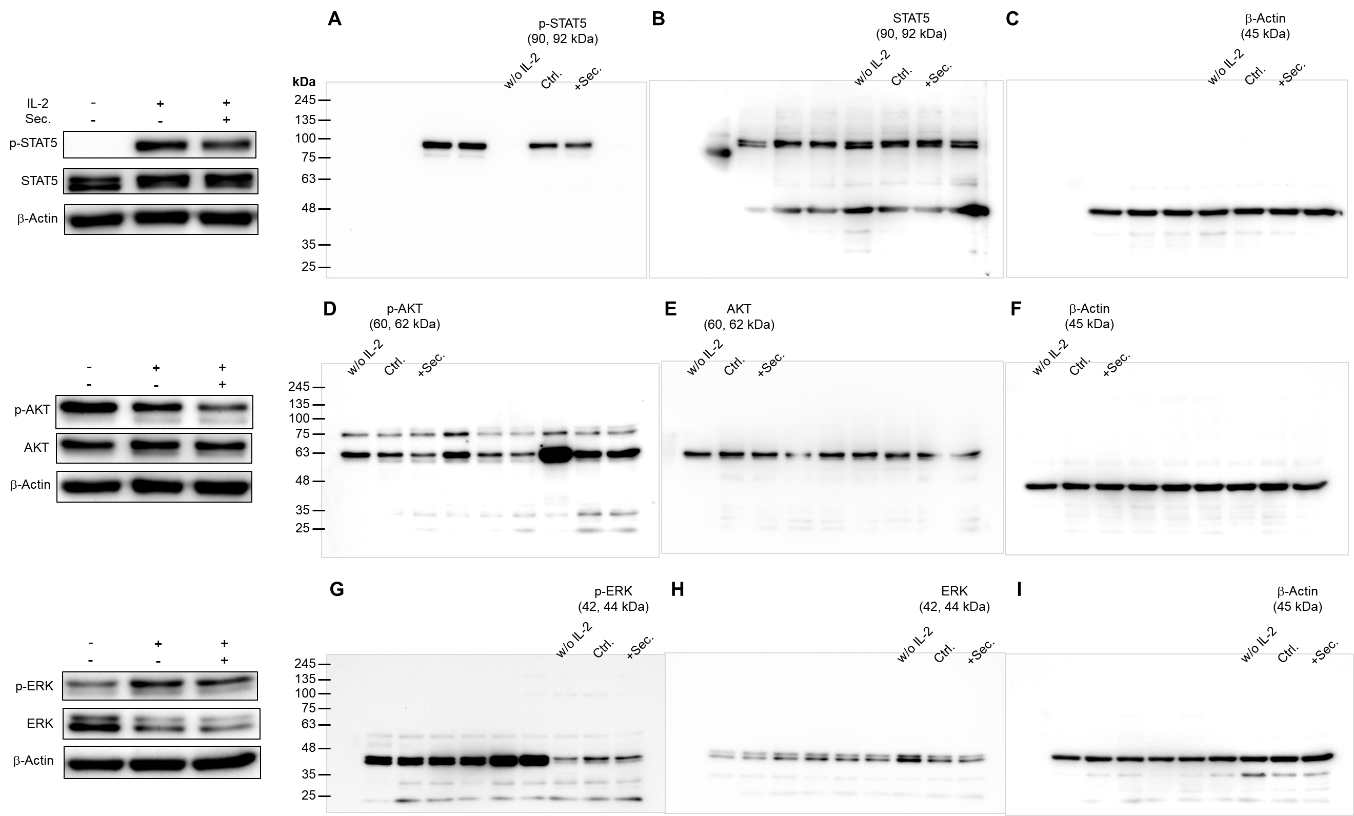


**Figure S4. Full-length blots of western blot images in figure 3B.**

(A: p-JAK1; B: JAK1; C: β-Actin; D: p-JAK3; E: JAK3; F: β-Actin)**
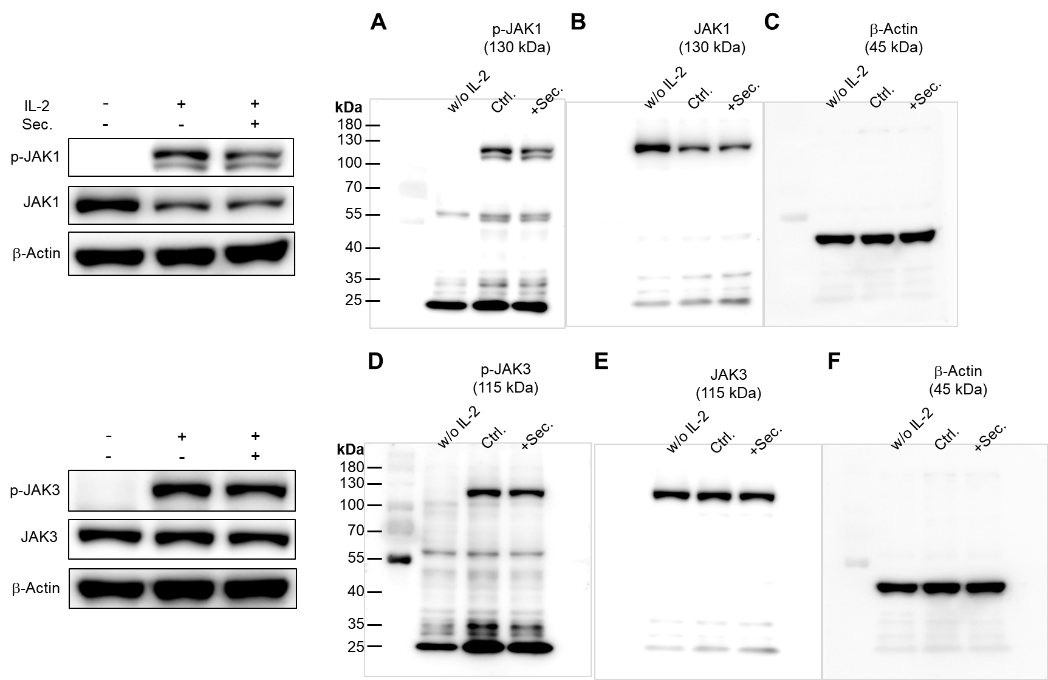
**

**Figure S5. Full-length blots of western blot images in figure 3C.**

(A: p-SHP1; B: SHP1; C: β-Actin; D: p-SHP2; E: SHP2; F: β-Actin; G: p-SHIP1; H: SHIP1; I: β-Actin)

**
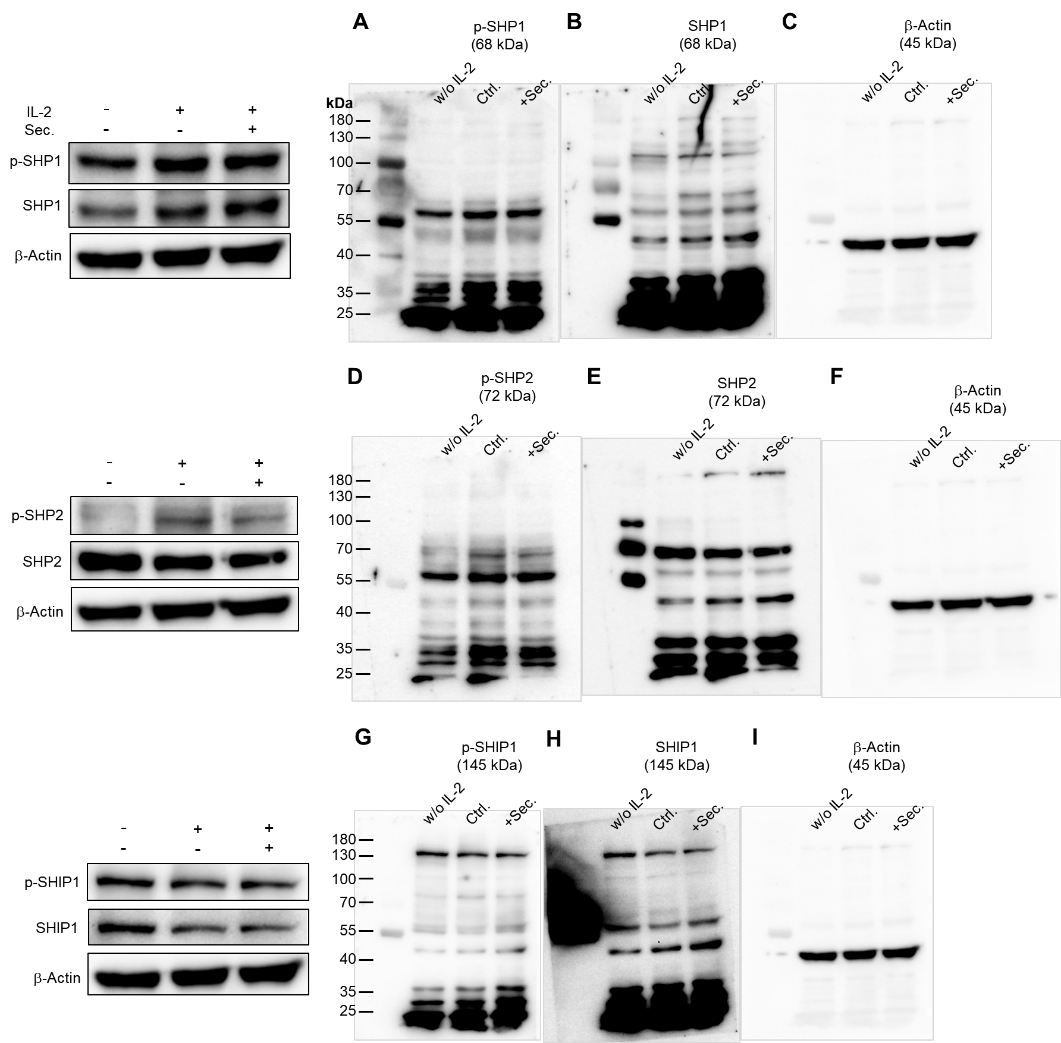
**

**Figure S6. Full-length blots of western blot images in figure 3D.**

(A: CIS; B: β-Actin)

**
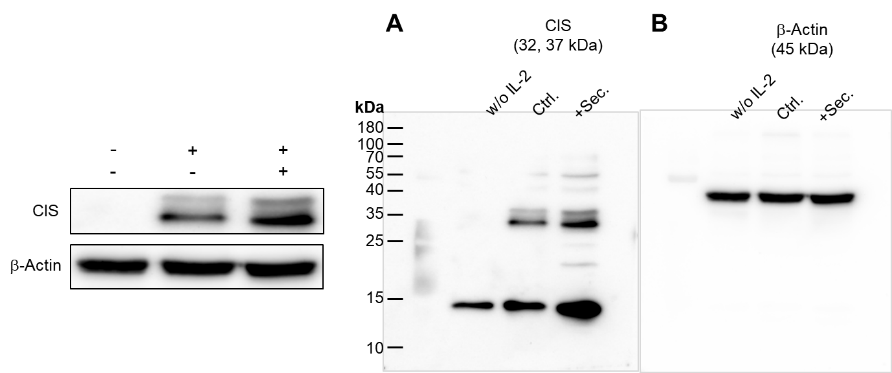
**

**Figure S7. Full-length blots of western blot images in figure 6E.**

(A: DUSP4 of donor 1; B: β-Actin of donor 1; C: DUSP4 of donor 2; D: DUSP4 of donor 3; E: β-Actin of both; F: DUSP4 of donor 4; G: β-Actin of donor 4; H: DTX1 of donor 1; I: β-Actin of donor 1)

**
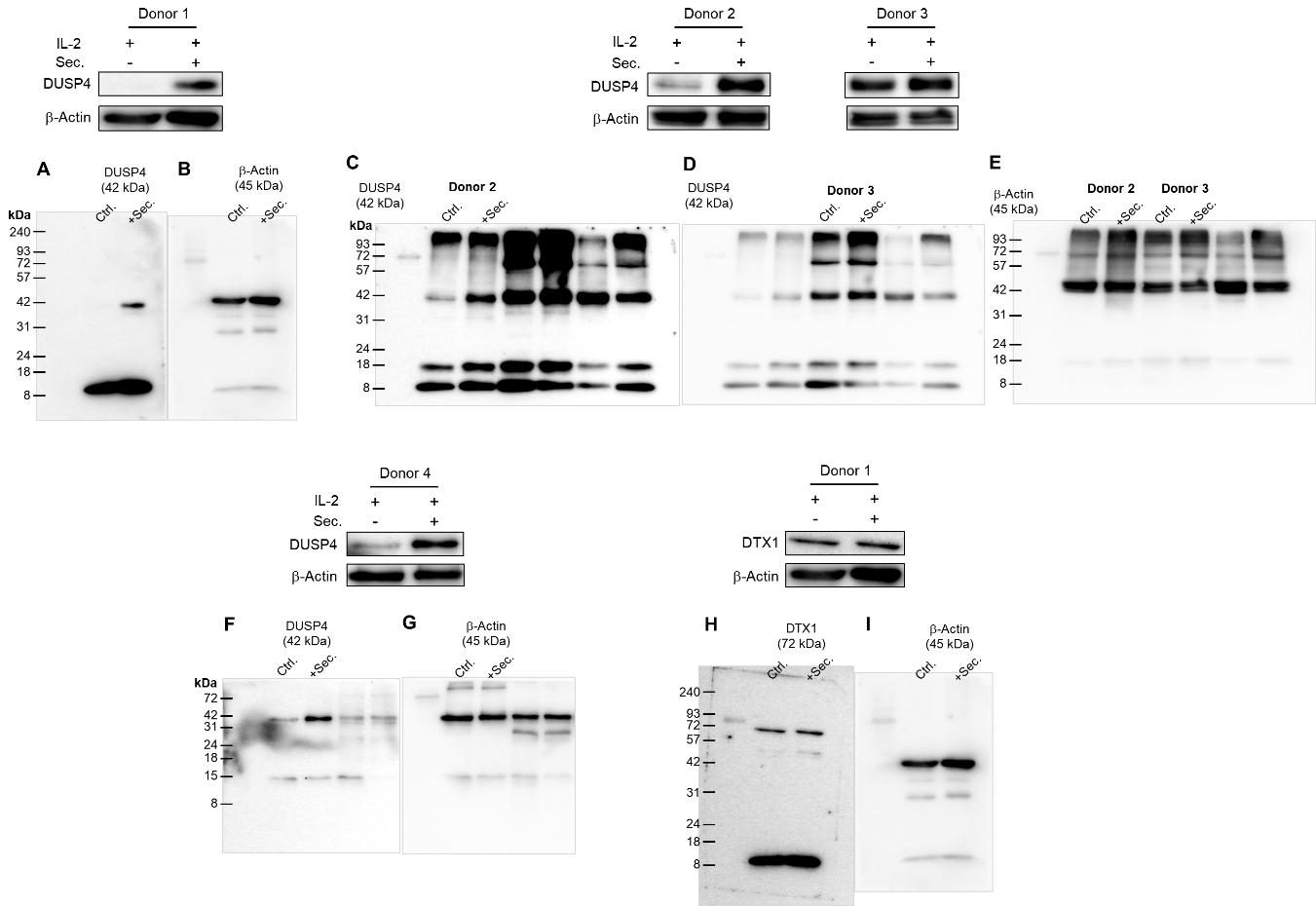
**
